# The molecular basis of thin filament activation: from single molecule to muscle

**DOI:** 10.1038/s41598-017-01604-8

**Published:** 2017-05-12

**Authors:** Thomas Longyear, Sam Walcott, Edward P. Debold

**Affiliations:** 1 Department of Kinesiology, University of Massachusetts, Amherst, MA 01003 USA; 2 Department of Mathematics, University of California, One Shields Ave., Davis, CA 95616 USA

## Abstract

For muscles to effectively power locomotion, trillions of myosin molecules must rapidly attach and detach from the actin thin filament. This is accomplished by precise regulation of the availability of the myosin binding sites on actin (i.e. activation). Both calcium (Ca^++^) and myosin binding contribute to activation, but both mechanisms are simultaneously active during contraction, making their relative contributions difficult to determine. Further complicating the process, myosin binding accelerates the attachment rate of neighboring myosin molecules, adding a cooperative element to the activation process. To de-convolve these two effects, we directly determined the effect of Ca^++^ on the rate of attachment of a single myosin molecule to a single regulated actin thin filament, and separately determined the distance over which myosin binding increases the attachment rate of neighboring molecules. Ca^++^ alone increases myosin’s attachment rate ~50-fold, while myosin binding accelerates attachment of neighboring molecules 400 nm along the actin thin filament.

## Introduction

At the molecular level, vertebrate striated muscle contraction is regulated by the actin binding proteins troponin (Tn) and tropomyosin (Tm) in a calcium- (Ca^++^-) dependent process. At the initiation of contraction, Ca^++^ is released from the sarcoplasmic reticulum and binds to the calcium binding subunit of Tn (TnC)^1^. TnC then undergoes a conformational change, exposing a hydrophobic patch to which the “switch-peptide” in the inhibitory subunit of Tn (TnI) binds, switching it from its inhibitory position on actin to TnC^2^. This motion allows Tm to relax further toward the groove between actin filament strands and results in the exposure of myosin binding sites on actin^3, 4^. Myosin then binds strongly to the thin filament (actin+Tn+Tm) resulting in muscle force generation and/or contraction.

Early work suggested that Ca^++^ caused the thin filament to behave as a binary switch, with Tm sterically blocking myosin binding and Ca^++^ causing the full azimuthal movement of Tm away from the myosin binding sites on actin^5^. However subsequent work demonstrated that Ca^++^ causes only a small increase in myosin’s affinity for actin^6^ and therefore does not directly regulate myosin binding. Indeed, structural studies of regulated thin filaments (RTFs) indicated that even at saturating Ca^++^ levels, Tm occupies a position on actin that only partially reveals the myosin binding sites on actin^3, 7^. Only after myosin strongly binds to actin are the binding sites fully revealed^7–9^. Thus both Ca^++^ and myosin strong binding play a role in the activation process, but their relative contribution remains unclear^10^.

Myosin binding is also responsible for causing the spread of thin filament activation. When one myosin binds to actin, the bound myosin increases the attachment rate of neighboring myosin molecules^9, 11–14^. This intermolecular coupling is evident at the cellular level by the steep sigmodial shape of the force-calcium relation in skinned muscle fibers, e.g. refs 15–19. The molecular basis for this coupling is that, when myosin binds strongly to actin, it displaces Tm and locally activates the RTF^9, 13, 20–23^. In addition to this type of intermolecular coupling, myosin molecules interacting with a common RTF are also mechanochemically coupled because forces generated by one motor affect the ADP release rate of other bound motors^24–27^. Although both types of intermolecular coupling are forms of cooperativity, activation of a RTF by myosin strong binding acts locally (on the scale of ~100 nm^28^), while mechanochemical coupling acts more globally (on the scale of ~10 *μ*m^27^). The distinction between these two types of cooperativity is important^29^, in part because a coupling distance must be defined for local coupling, while global coupling equally affects each myosin molecule interacting with a RTF. Therefore to gain a complete understanding of the molecular basis of the role of Ca^++^ in muscle activation, two questions must be answered: 1) how does Ca^++^ affect myosin binding to a RTF at the single molecule level? and 2) how does Ca^++^ affect the coupling between nearby myosin molecules? To answer these questions, we performed measurements of myosin’s interaction with RTFs at a range of Ca^++^ concentrations using single molecule^30^ and mini-ensemble (~14 independent myosin heads)^31, 32^ laser trap assays, and using the *in vitro* motility assay with a large myosin ensemble (~75 independent myosin heads). Since each of these experiments was performed at a different myosin surface density, the coupling between molecules was varied, from no coupling (single molecule laser trap), to weak coupling (small ensemble laser trap) to strong coupling (motility).

## Results

### The Ca^++^-dependence of a single myosin molecule interacting with a single regulated thin filament

**Figure 1:**
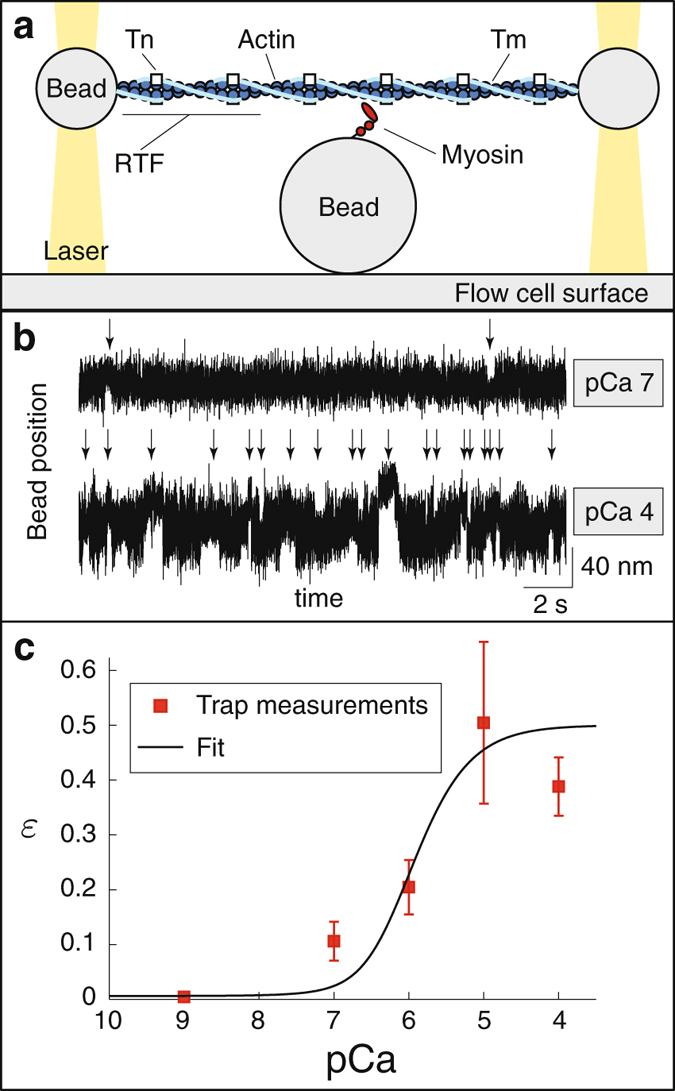
Direct measurements of the effect of Ca^++^ on the rate at which a single myosin molecule binds to a regulated thin filament (RTF). (**a**) Cartoon of the single molecule laser trap (not to scale). (**b**) Raw data, showing the position of one of the laser-trapped beads as a function of time. Binding events, indicated with a vertical arrow, are much less frequent at low Ca^++^ (pCa 7) than at high Ca^++^ (pCa 4). (**c**) Event frequency, scaled by the event frequency in the absence of regulation (2.2 s^−1^) as a function of Ca^++^ is well-fit by equations (1) and (2), with *K* = 0.216 *μ*M. Symbols indicate mean frequency, error bars show standard deviation. The data point at pCa 9 is from ref. 28. ATP concentration is 1 *μ*M.

In the single molecule laser trap assay with an actin filament and no regulatory proteins, we observed an average event frequency of 2.2 s^−1^ (594 binding events over 270 s). When we performed this assay with an actin filament including regulatory proteins (Fig. 1a), single myosin molecules bound to the RTF at a frequency of 0.85 ± 0.33 s^−1^ (mean ± SD) at saturating Ca^++^ (pCa 4, where pCa is the negative log_10_ of the calcium concentration). This experiment was then repeated at progressively lower Ca^++^ levels (pCa 5, 6, 7 and 9). An obvious decrease in binding frequency (Fig. 1b) was observed, with little effect on attachment lifetimes and unitary displacements (see Supplementary Material, SM). In fact, binding events were so infrequent at pCa 9 (<0.01 s^−1^) that it was difficult to generate a sample size that could yield a confident estimate of the average frequency. We therefore include a previously published value for binding frequency at pCa 9^28^, which suggests a 50-fold reduction from pCa 4 (Fig. 1c).

In these experiments, event frequency is ~20-fold slower than the weak-to-strong binding transition measured in solution (40 s^−1^ refs 33, 34), because fluctuations in RTF height effectively reduce the concentration of actin. Differences in RTF height could therefore lead to variability in attachment rate, so we determined the height of the RTF above the myosin coated pedestal to ensure that variability in this height did not confound or influence the rate of myosin attachment to the RTF in a manner independent of Ca^++^. The previously published measurements at pCa 9^28^ were collected without monitoring RTF height, but our measurements suggest that this height does not vary with pCa (see Fig. S1). Since all data were collected at a similar RTF height and therefore at the same effective actin concentration, measuring the attachment rate relative to the attachment rate in the absence of regulation (Fig. 1c) removes the effect of actin concentration (see SM).

### The Ca^++^-dependence of a mini-ensemble of myosin molecules interacting with a regulated thin filament

**Figure 2:**
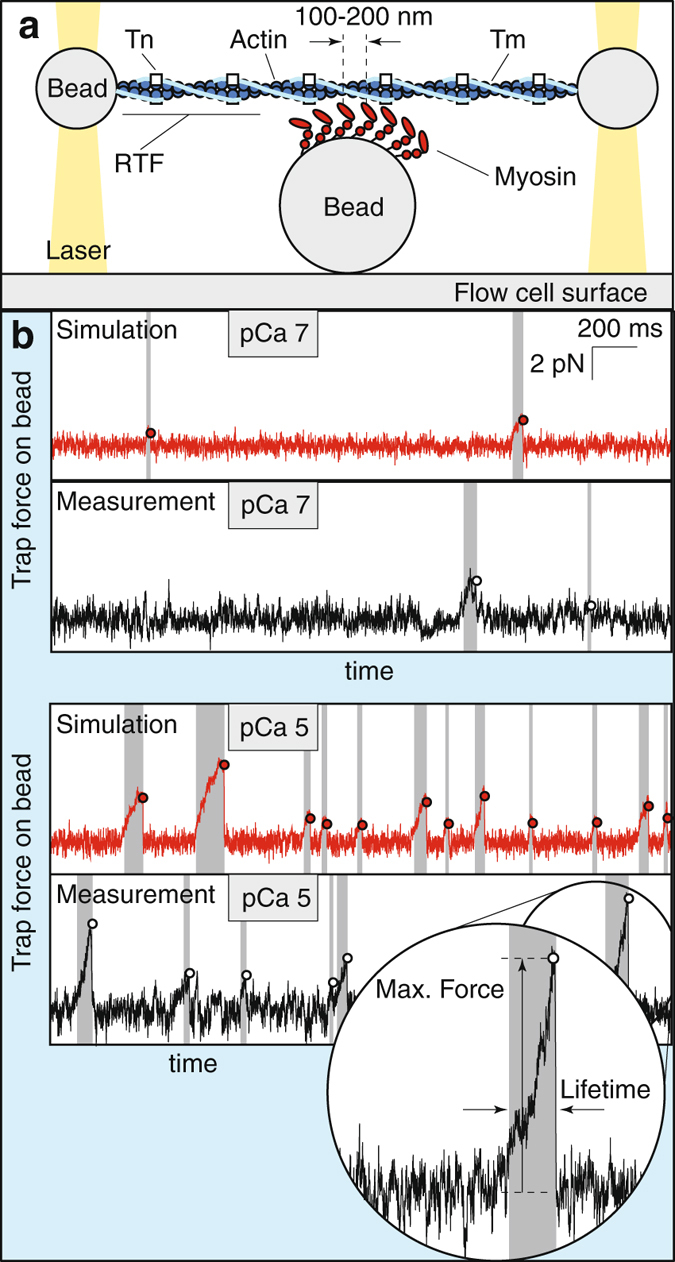
Ca^++^ affects the maximum force, duration and frequency of binding events from a mini-ensemble of myosin interacting with a regulated thin filament (RTF). (**a**) Cartoon of the mini-ensemble laser trap (not to scale). (**b**) Measured (black) and simulated (red) data, showing the force applied by the laser trap on one of the beads as a function of time. Binding events (shaded) are less frequent, shorter, and reach a smaller maximum force (hollow dot) at low Ca^++^ (pCa 7) than at high Ca^++^ (pCa 5). Inset shows how our custom event detection algorithm identifies maximum event force and lifetime. For these traces, trap stiffness was 0.0298 pN/nm (pCa 7) and 0.0382 pN/nm (pCa 5). ATP concentration is 100 *μ*M.

**Figure 3:**
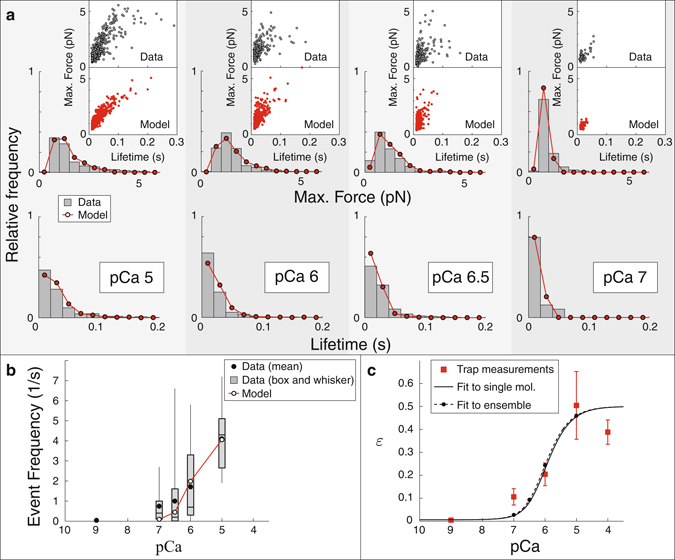
The model reproduces the Ca^++^-dependence of the maximum force, lifetime and frequency of binding events measured using a laser trap with a mini myosin ensemble. (**a**) The model reproduces the distribution of maximum forces (top) and lifetimes (bottom) of binding events at four different Ca^++^ concentrations. In each plot, histograms from the model (hollow circles) are superimposed over histograms from our measurements (gray). Each inset shows the maximum force and lifetime of every event as a dot. The model (bottom, red) reproduces the correlation between these two quantities observed in our measurements (top, blue). (**b**) The model reproduces the frequency of binding events. Data, measured over a 10 s interval, are shown as box and whisker plots, the average event frequency is shown for the model (hollow circles). Note that, because of the temporal resolution of the laser trap, the binding frequency is likely an underestimate of the true frequency. This effect is taken into account in the model to ensure a fair comparison between simulation and measurement. (**c**) Single molecule myosin binding frequency (*ε*), predicted by fitting the model to the mini-ensemble measurements (dashed line), agrees with our direct, single molecule trap measurements (data, red squares; error bars, SD; best fit, solid line). Parameters used in these simulations were *N*_*M*_ = 14, *k*_*a*2_ = 36 s^−1^ and *K* = 0.2 *μ*M. ATP concentration is 100 *μ*M.

To provide insight into the coupling between myosin molecules, we increased the concentration of myosin in solution from 0.2 *μ*g/mL to 10 *μ*g/mL in the laser trap assay (Fig. 2a). These mini-ensembles of myosin generated large displacements of the RTF at near-saturating Ca^++^ (pCa 5), consistent with multiple myosin molecules successively binding to and displacing the RTF (Fig. 2b). The size, lifetime and frequency of the displacements generated in the mini-ensemble assay decreased as Ca^++^ was lowered until, at pCa 9, the size of the displacements was consistent with mostly single myosin binding events (9.3 ± 1.4 nm, mean plus/minus SEM, not significantly different from single molecule displacement measurements at pCa 7, *p* > 0.05, Mann-Whitney rank sum test). Because the laser trap behaves as a linear spring, the trap stiffness was used to convert the displacements from binding events into forces^35^ (Fig. 3a). The average peak force was significantly lower as [Ca^++^] decreased, except pCa 5 vs. 6 and pCa 6.5 vs. 7 (see SM), and the event duration was significantly lower at each successive [Ca^++^], except pCa 5 vs. 6.5 (see SM). Average event frequency also decreased strongly with decreasing [Ca^++^], from 4.1 events/s at pCa 5 to 0.05 events/s at pCa 9 (Fig. 3b). Note that, because of the temporal resolution of the laser trap, this binding frequency is likely an underestimate and does not directly reflect the attachment rate.

### Model

Because the binding of one myosin molecule locally activates the thin filament, by increasing the density of myosin in our laser trap assay, we introduce local coupling between the myosin molecules. Varying this local coupling allowed us to separate the effect of Ca^++^ on myosin’s interaction at the single molecule level from its effect on the coupling between myosin molecules. This was accomplished using a model that incorporates a four-state kinetic model of myosin’s interaction with unregulated actin that includes global coupling between molecules, and describes single molecule, small ensemble and motility measurements^27, 32^ – the same experiments performed here in the presence of regulation. We did not change any model parameters, except the ATP-dependent detachment rate *k*_*T*_, which we increased from *k*_*T*_ = 2 *μ*M^−1^ s^−1^ to *k*_*T*_ = 3 *μ*M^−1^ s^−1^, based on our *in vitro* motility measurements (see SM).

Regulation is modeled with a simplified version of the continuous flexible chain model^21, 22, 36^, which is defined by two parameters^37, 38^: 1) *ε*(Ca^++^), the single molecule weak-to-strong binding transition rate as a function of [Ca^++^] relative to that rate in the absence of regulation; and 2) $\ell$, a coupling distance, such that two myosin molecules, strongly bound to a RTF, completely activate the intervening filament if separated by a distance less than $\ell$ and act independently if separated by a distance greater than $\ell$. Note that this distance is not equivalent to the size of a cooperative unit^9^, since the binding of one myosin only partially activates the thin filament (see SM).

Thus, in the model, the effect of Ca^++^ on myosin’s interaction at the single molecule level is given by *ε*(Ca^++^) and its effect on the coupling between myosin molecules is given by $\ell$. We used $\ell =400\,{\rm{nm}}$ ^29, 37, 39^ (see SM for a discussion of how this distance relates to the size of a cooperative unit), and assumed that this distance is independent of Ca^++^. We then varied *ε*(Ca^++^) to fit the model to the data. To make this problem tractable, we assume that *ε*(Ca^++^) takes the following form ref. 38 (see SM for a derivation):

1 $$\varepsilon ={\varepsilon }_{min}^{1-\theta }{\varepsilon }_{max}^{\theta }$$

where *θ* is the fraction of Tn that has bound Ca^++^. From previous work (see SM), we estimate *ε*_*max*_ = 0.5 and *ε*_*min*_ = 0.006^28, 29, 37^. We assume Michaelis-Menten saturation of Tn with Ca^++^^38^ (see SM for alternate models)

2 $$\theta =\frac{[{{\rm{Ca}}}^{++}]}{K+[{{\rm{Ca}}}^{++}]}$$

With these assumptions, there is only a single parameter that defines *ε*(Ca^++^), *K*, TnC’s affinity for Ca^++^ in the absence of myosin strong binding to the RTF.

### Comparing single molecule and mini-ensemble measurements

In the single molecule laser trap, there is no coupling between myosin molecules, so the data show the effect of Ca^++^ on the weak to strong binding transition rate. However, the measured rate is not directly the weak to strong binding transition, because fluctuations in the height of the actin filament effectively lower the actin concentration. Since identical fluctuations occur in all measurements, by dividing our measurements by this unregulated binding rate, 2.2 s^−1^, we obtain the relative weak to strong binding transition, given by model parameter *ε*(Ca^++^). Our data are well-fit by equations (1) and (2) (fits not significantly different from data, *p* > 0.05, *χ* ^2^ test, Fig. 1c), and give an estimate of *K* = 0.216 ± 0.055 *μ*M (median plus/minus one quartile, estimated by bootstrapping, see SM).

In the mini-ensemble laser trap measurements, there is coupling between molecules. We used the model to separate the effects of this coupling from the effect of Ca^++^ on myosin’s weak to strong binding transition. Doing so required the specification of two unknown parameters, *N*_*M*_, the number of myosin molecules capable of interacting with the actin filament, and *k*_*a*2_, defined as follows. As discussed previously, the binding of a myosin molecule to actin in the laser trap is slowed by fluctuations in filament height (*z*). However, if a mini-ensemble of myosin is present, after the first myosin molecule binds, subsequent myosin molecules attach to actin at a rate that is closer to the weak-to-strong binding transition rate, likely due to the bound myosin molecule restricting the fluctuations in *z*. In previous simulations of mini-ensemble measurements in the laser trap^32^, this effect was modeled by assuming that the attachment rate of the second myosin, *k*_*a*2_, had an intermediate value between the single molecule binding rate (2.2 s^−1^) and the weak-to-strong binding transition (40 s^−1^ refs 33, 34). After the second myosin molecule bound, subsequent myosin molecules attach at the weak-to-strong binding transition rate of 40 s^−1^ ^32^.

Given the identical assay geometry, in our simulations of myosin ensembles interacting with a RTF we assume that similar effects occur, and we determined the values of *k*_*a*2_ and *N*_*M*_ by fitting the model to our measurements at near-saturating Ca^++^ (pCa 5). This fitting procedure involved simulating raw data, as shown in Fig. 2b, and then analyzing it with the same customized programs used to analyze our measurements, as described in Methods (also see SM). Based on these fits, we estimate that there are between *N*_*M*_ = 14 and *N*_*M*_ = 22 independent myosin heads in these small ensemble measurements. This estimate is in broad agreement with an independent estimate of *N*_*M*_ = 7 based on assay geometry and myosin surface density^31, 40^.

We inferred the relative weak to strong binding transition, *ε*(Ca^++^), from these mini-ensemble measurements. To do so, we used the same procedure to compare the model to the remaining data collected at sub-saturating Ca^++^ (pCa 6, 6.5 and 7). We optimized the fit of the model to the data by varying the parameter *K* in equations (1) and (2). The model reasonably replicates the maximum force and event lifetimes of our experimental measurements (Fig. 3a, see Fig. S8 for averages), and also reasonably replicates our event frequency measurements (Fig. 3b). Regardless of the value of *N*_*M*_, the best estimate of *K* was between 0.15 and 0.20 *μ*M (see SM). Based on previous modeling of small ensemble trap measurements, where an estimated 16–20 independent heads were available to interact with actin^27, 32^, we expect that *N*_*M*_ is closer to 14, since the myosin concentration is lower in these current experiments (10 *μ*g/mL, compared to 15 *μ*g/mL). For this ensemble size, *K* = 0.199 ± 0.044 *μ*M (mean plus/minus SD), in good agreement with our direct single molecule measurements (Fig. 3c).

### The Ca^++^-dependence of a large ensemble of myosin molecules interacting with a regulated thin filament

**Figure 4:**
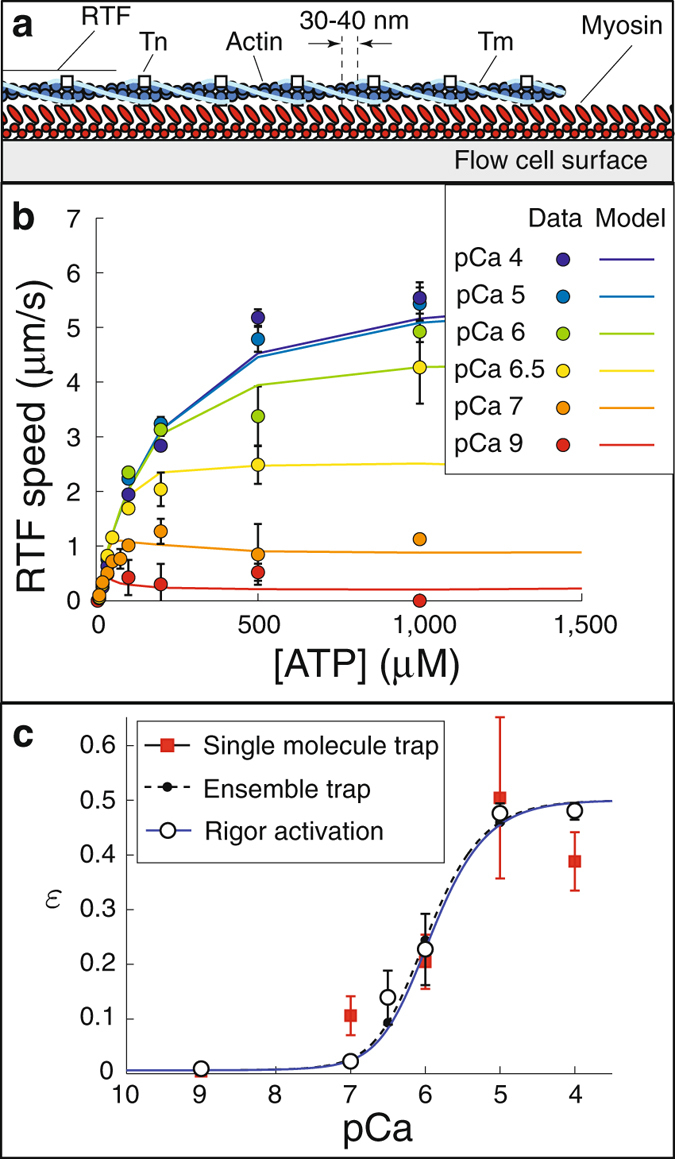
Measurements of the effect of Ca^++^ on the ATP-dependence of the speed of regulated thin filaments (RTFs) in the *in vitro* motility assay. (**a**) Cartoon of the *in vitro* motility assay (not to scale). (**b**) The model reproduces the Ca^++^-dependence of RTF speed as a function of ATP. Note the biphasic behavior at low Ca^++^ (pCa 7 and 9). Data (circles) are means, error bars show S.E.M. Parameters used in the simulations were *N*_*mot*_ = 75, *K* = 0.217 *μ*M. (**c**) Single molecule myosin binding frequency (*ε*), predicted by fitting the model to motility measurements at each Ca^++^ concentration (hollow circles, error bars show S.D.), agree with our direct, single molecule measurements (data, red squares; error bars, S.D.; best fit, black solid line) and our indirect estimate from fits to the mini-ensemble measurement (dashed line). These individual fits to the *in vitro* motility data are well-described by equations (1) and (2), with *K* = 0.217 *μ*M (blue solid line).

Independently, the single molecule and small ensemble measurements give a consistent description of how Ca^++^ regulates the binding of a single myosin molecule to a RTF. This result suggests that we have both accurately characterized the Ca^++^-dependence of myosin attachment, *ε*(Ca^++^), and the spread of activation upon myosin attachment with $\ell$. To further test these hypotheses, we performed a series of experiments in the motility assay at a myosin concentration that saturates the surface (100 *μ*g/mL). This results in roughly a five-fold increase in myosin density on the surface, and therefore a five-fold increase in coupling compared to the mini-ensemble measurements (Fig. 4a).

Under these strongly coupled conditions, when strong binding is prolonged by lowering the ATP concentration and thereby extending the lifetime of the rigor state, myosin can bind to and activate the thin filament even in the absence of Ca^++^^11, 28^. In a solution lacking Ca^++^ and with very low ATP, average RTF speed (*v*) in the motility assay increases linearly with ATP because the RTFs are activated by myosin binding in rigor and because myosin’s detachment rate from a RTF, which is proportional to ATP at sufficiently low ATP concentrations, determines *v* under these conditions^27, 37, 41^. However, above a critical ATP concentration, *v* abruptly decreases with increasing ATP because strong binding lifetime is no longer sufficient to activate the RTF^28^. Previously, such data have provided precise estimates of *ε* in the absence of Ca^++^^37^; so to further test our hypotheses, we repeated these experiments at a range of Ca^++^ (pCa 4, 5, 6, 6.5, 7, and 9). Consistent with previous measurements^28^, we observed Ca^++^-independent activation below a critical ATP concentration (~60 *μ*M); but the effect of Ca^++^ becomes clear at higher ATP concentrations (Fig. 4b).

We used the mathematical model to compare these measurements to the mini-ensemble and single molecule laser trap measurements. To do so, we estimated the number of myosin molecules available to interact with the RTF, *N*_*mot*_ = 75, based on fitting the model to our measurements at high Ca^++^ (an increase from previous estimates in the absence of regulation^27^, perhaps due to an increase in filament stiffness due to the regulatory proteins^42^, see SM). Then, to fit the data, at each Ca^++^ concentration, we determined the value of *ε* that optimized the model fit (see SM for fits and sensitivity analysis). In this way, we obtained an estimate of *ε*(Ca^++^) that is independent of our assumed form of equations (1) and (2). The resulting data points are well-fit by a curve of the form of equations (1) and (2), with *K* = 0.217 ± 0.034 *μ*M (*p* > 0.05, *χ* ^2^ test, mean plus/minus SD). Using these parameters, the model reasonably captures our measurements (Fig. 4b).

This estimate of *ε*(Ca^++^), along with the fit, is remarkably consistent with our two previous, independent estimates based on direct single molecule measurements and model fits to the small ensemble trapping experiments (Fig. 4c, pairwise comparisons of estimates not sig. different; *p* > 0.05, t-test). This consistency demonstrates that the model successfully separates the Ca^++^-dependent and myosin-dependent contributions to thin filament activation. Thus, we have not only precisely measured how Ca^++^ contributes to activation (Fig. 4c), but also, via the model, provided a detailed description of how myosin-binding contributes to activation.

## Discussion

Activation of striated muscle requires both a Ca^++^-dependent increase in the rate myosin binds to a RTF and a myosin binding-induced local activation of the RTF. The first of these changes occurs at the single molecule level, while the latter occurs only when multiple molecules interact with a RTF. Thus, a complete understanding of the molecular basis of thin filament activation must both span the single molecule and ensemble scales and de-convolve these two activation processes. We have accomplished this task by 1) directly measuring Ca^++^-dependent single molecule binding of myosin to a RTF; 2) performing two additional assays at low and high myosin densities, which affects the degree of myosin binding-induced activation but not Ca^++^-dependent activation; and 3) using a mathematical model to de-convolve the two effects. The consistency of the single molecule Ca^++^-dependent activation across all three assays (Fig. 4c) demonstrates that we have successfully separated the two activation processes and provides, for the first time, a detailed description of both.

Our observations of Ca^++^’s effect on single myosin binding to RTFs, both our direct measurements (Fig. 1) and our two indirect measurements interpreted with the model (Figs 3 and 4), confirm that Ca^++^ regulates myosin’s weak to strong binding rate in a dose-dependent manner^1^. Even at saturating Ca^++^, the presence of regulatory proteins reduces event frequency from 2.2 s^−1^ to 0.85 s^−1^, suggesting that the regulatory proteins introduce an energy barrier that reduces strong binding two-fold, consistent with recent direct observations of fluorescently labeled myosin binding to RTFs^12, 29^. This event frequency progressively decreases to just 0.02 s^−1^ in the absence of Ca^++^ (pCa 9), suggesting that Ca^++^ alone can increase the probability of strong binding nearly fifty-fold. One of our central results is that we have characterized the Ca^++^-dependence of strong binding over the physiological range of Ca^++^ concentrations. Assuming that Tn binds Ca^++^ according to Michaelis-Menten kinetics, and given the energy barriers measured at saturating Ca^++^ and in the absence of Ca^++^, these measurements should be well-fit by equations (1) and (2). Consistent with this expectation, these equations fit all of our data and give an estimate of $K\approx 0.2\,\mu {\rm{M}}$ for the Michaelis-Menten constant of Tn binding Ca^++^. Intriguingly, and in support of this estimate of *K*, this saturation curve agrees with direct measurements of Ca^++^ binding to Tn in muscle fibers when myosin binding is eliminated (see SM).

**Figure 5:**
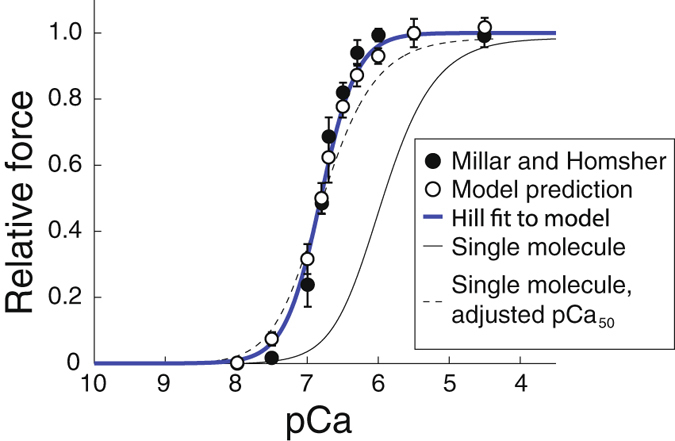
Strong-binding activation is necessary and sufficient to fit fiber data with the model. If strong-binding activation can be neglected, then the isometric force developed by a muscle fiber, which is proportional to the number of strongly bound myosin, should be described by the same curve as single molecule data. The response of a single myosin molecule to calcium (thin black line, pCa_50_ = 5.96, *α* = 1.4 ± 0.3, mean plus/minus SD) is inconsistent with measurements of isometric force as a function of Ca^++^^18^ (filled circles, error bars show SEM). Even when the pCa_50_ of the single molecule curve is adjusted to match the measurements (dashed black line), the curve is not steep enough to fit the data. When strong-binding activation is included in the model (hollow circles, error bars show SD), simulation results are consistent with the measurements since a Hill fit to the model (heavy blue line, pCa_50_ = 6.81 ± 0.03, *α* = 1.71 ± 0.19, mean plus/minus SD) also fits the measurements. ATP concentration 4000 *μ*M.

Although it is generally agreed that myosin strong binding contributes to thin filament activation at low ATP^1^, there is some controversy about whether this occurs at physiological ATP^10^. We can test whether myosin strong binding contributes to activation by comparing the single molecule activation curve we measured to the isometric force developed in muscle fibers at different Ca^++^ concentrations (a force-pCa curve) at physiological ATP. If each myosin molecule acts independently in a cell, i.e. if strong-binding activation can be neglected, then the isometric force developed by a muscle fiber, which is proportional to the number of strongly bound myosin, should be described by the same curve as single molecule data. Fitting our single molecule activation measurements with a Hill equation gives a Hill coefficient of *α* = 1.4 ± 0.3 (mean plus/minus SD). This Hill coefficient is too low to describe most force-pCa curves collected at physiological ATP (e.g. refs 15–19) and, consistent with this result, when our measured binding frequency-Ca^++^ curve is compared to isometric force-Ca^++^ data^18^, the latter curve is clearly steeper and shifted to the left (Fig. 5). The difference between the two curves suggests the existence of myosin strong-binding induced activation, even at physiological ATP.

To characterize the contribution of myosin binding to the activation of the RTF, we performed experiments at increased myosin density, which introduces local coupling between myosin molecules, first in the mini-ensemble laser trap assay (Fig. 2) and then in the motility assay (Fig. 4). By increasing the myosin concentration in the laser trap assay (Fig. 2a) we increased the myosin heads available to interact with the thin filament from one to ~14. At saturating Ca^++^ this increase resulted in large runs of motility, indicating that multiple myosin molecules were simultaneously bound to the thin filament (Fig. 2a). As Ca^++^ was decreased, the number of these long runs decreased, as did the frequency of binding events (Fig. 3). In our mathematical model, coupling between myosin molecules is defined by the length scale $\ell$, which has been previously estimated to be 400 nm^29, 37, 39^. With this value, our mathematical model reproduces our observations and predicts a Michaelis-Menten constant for Tn binding Ca^++^ (*K* = 0.199 ± 0.044 *μ*M) nearly identical to what we measured in our single molecule experiments (*K* = 0.216 ± 0.055 *μ*M, Fig. 3c). Remarkably, in the motility assay when myosin density was increased five-fold and ATP concentration was lowered (Fig. 4), both of which increase the coupling between myosin molecules, our mathematical model reproduces our observations and predicts a Michaelis-Menten constant for Tn binding Ca^++^ (*K* = 0.217 ± 0.034 *μ*M) that is nearly identical to what we measured in our single molecule and small ensemble experiments (*K* = 0.199 ± 0.044 *μ*M and *K* = 0.216 ± 0.055 *μ*M, respectively, Fig. 4c). Thus, all of our data, regardless of the coupling between myosin, can be explained by the same Ca^++^-dependent single molecule activation curve (given by equations (1) and (2) with $K\approx 0.2\,\mu {\rm{M}}$) and the same Ca^++^-independent coupling distance, $\ell =400\,{\rm{nm}}$.

Because the estimate of $\ell =400\,{\rm{nm}}$ was derived previously, based on different data sets^29, 37, 39^, we performed a separate analysis based on the present data to determine this value again. This was done by varying $\ell$ in our model and comparing the model’s predictions to our measurements. The mini-ensemble laser trap and motility measurements independently predict a coupling distance of $\ell =400\,{\rm{nm}}$; in addition, all three data sets are consistent only if $\ell =400\pm 100\,{\rm{nm}}$ (see SM). Thus, a Ca^++^-independent $\ell =400\,{\rm{nm}}$ is both sufficient and necessary to explain our data. This result suggests that we have correctly characterized the spread of activation along the thin filament.

This coupling distance $\ell =400\,{\rm{nm}}$ is larger than previous estimates of what others characterize as a cooperative unit, *u* ^28, 43^. The reason for the difference between $\ell$ and *u* is that *u* is based on a model that assumes the binding of one myosin completely activates the thin filament over the distance *u* ^9^; while, in our model, the binding of a single myosin generates partial activation over the distance $\ell$. Because of this difference, the relationship between *u* and $\ell$ depends on experimental details. For example, we have previously demonstrated that a coupling distance of $\ell =400\,{\rm{nm}}$ gives an effective cooperative unit of *u* = 107 nm in the laser trap^37^, which compares well with the measured value of *u* = 111 nm^28^. Additionally, observations of fluorescently-tagged myosin binding to RTFs that predict a cooperative unit spanning 11 binding sites (*u* = 11 · 5.1 nm = 57 nm, ref. 12) are consistent with the model presented here with a coupling distance of $\ell =400\,{\rm{nm}}$ ^29^. Thus our findings are consistent with previous estimates of the distance along a RTF over which myosin binding accelerates the binding of neighboring myosin molecules. Further, since our model explains these data with a single coupling distance ($\ell =400\,{\rm{nm}}$), while the cooperative unit varies nearly by a factor of two (*u* = 111 nm^28^ and *u* = 57 nm^12^), we add support to the view that myosin binding generates partial, as opposed to full, activation of the thin filament, e.g. ref. 23 (see ref. 37 for further discussion and SM for a mathematical description).

Our molecular-scale experiments allow us to define activation at the molecular level, but how do they translate to the level of a whole muscle, where trillions of myosin molecules act collectively to generate force and motion? Given *K* = 0.2 *μ*M in equations (1) and (2) and $\ell =400\,{\rm{nm}}$, the model has no free parameters and can therefore be used to predict contractile properties at the muscle fiber scale. Scaling up the simulations to reflect the number of molecules in the half sarcomere (~300, ref. 44) and assuming spacing between adjacent myosin molecules similar to the motility assay allows the model to predict the force-Ca^++^ relation in a fiber. When we performed these simulations, we observed clear differences between the predicted force-pCa curve (Hill coefficient *α* = 1.71 ± 0.19, pCa_50_ = 6.81 ± 0.03, mean plus/minus SD) and our single molecule binding frequency measurements (*α* = 1.4, pCa_50_ = 5.96, mean plus/minus SD, Fig. 5). The increase in both *α* and pCa_50_ in the half sarcomere simulations show that, in the model at physiological ATP, myosin molecules working together generate more force than when working in isolation because myosin strong-binding activates the RTF; moreover, this strong-binding activation is sufficient to explain differences between single molecule and fiber activation, since the model predicts an isometric force-pCa curve that is consistent with previous measurements^18^ (Fig. 5, a single Hill curve describes both the data and model simulations, *p* > 0.05, *χ* ^2^-test). This approach can also be used to address longstanding controversies in the field (e.g. besides finding support for our estimate of *K* ^15^, we also find support for the hypothesis that myosin strong binding to a RTF activates it, and enhances Ca^++^ binding to Tn^15, 19^, see SM). This suggests that the molecular basis of activation we elucidated at the single molecule and small ensemble levels underlies the collective behavior of the trillions of myosin molecules in a muscle fiber. It is, however, worth noting that fiber force data includes contributions from non-myosin, Ca^++^-sensitive proteins, like titin^45^ and myosin binding protein C^46, 47^, which we neglect. Together, the model and measurements provide a consistent description of muscle activation from the scale of a single molecule to the scale of a cell, a description that has, until now, remained elusive.

## Materials and Methods

### Proteins

Chicken skeletal actin and myosin were isolated as previously described^48^ with minor modifications^49^. Isolated troponin complexes from rabbit fast skeletal muscle and tropomyosin from cardiac muscle, were obtained from Life Diagnostics Inc. (West Chester, PA). Thin filaments were reconstituted by mixing 1 *μ*M biotin/TRITC-labeled actin filaments with 0.25 *μ*M Tn and 0.25 *μ*M Tm and incubating at 4 C for 3 hours prior to experimentation, as previously described^28^. Complete regulation of actomyosin binding was ensured by the absence of filament motion in a motility assay in the absence of free Ca^++^ (pCa 10).

### *In vitro* motility assay

The *in vitro* motility assay using RTFs were performed as previously described^50^, with minor modifications. Briefly, myosin was loaded onto a nitrocellulose-coated coverslip surface at a saturating concentration of 100 *μ*g/mL. The surface was then blocked with BSA for 1 minute, an actin coat was added as another precaution to eliminate deadheads. TRITC labeled actin was added and allowed to incubate for 1 minute. 0.75 *μ*M Tn and 0.25 *μ*M Tn were then added and allowed to incubate for 7 minutes to form RTFs as previously detailed^51^. Finally, a low salt buffer was added to wash out proteins not bound to the surface and then the final buffer was added, containing a range of calcium and ATP concentrations. RTF motion was visualized using a Nikon Ti-U inverted microscope, with a 100x, 1.4 NA CFI Plan Apo oil-coupled objective with the temperature maintained at 30.0 °C for all experiments. For each flow cell, three 30 s videos were captured at 10 frames/s and at three different locations within each flow cell.

### Analysis of motility data

The velocity of the RTFs was determined using an automated filament-tracking ImageJ plugin WRMTRK. In an effort to eliminate the possibility of analyzing noise in the fluorescence signal, filaments shorter than 0.5 *μ*m were eliminated from the analysis, and filaments with velocities less than 0.13 *μ*m/s were considered to be stationary. A typical field of view generated 25–75 filament velocities, and the mean of these velocities was taken as the average velocity for that field of view. The microscope slide was then moved to a new field of view within the flow cell twice more to generate a total of three recordings for each flow cell. For each condition tested, at least three flow cells were used to generate the data, resulting in at least nine recordings contributing to the overall mean filament velocity for each condition.

### Laser trap assay

The laser trap assays were performed as previously described^32^ adjusting the myosin concentration to yield either single molecule (0.2 *μ*g/mL) or mini-ensemble (10 *μ*g/mL) binding events. Following a blocking of the surface with bovine serum albumin (0.5 mg/mL), the low-salt experimental buffer (25 mM KCl, 25 mM imidazole, 1 mM EGTA, and 4 mM MgCl_2_) with 100 *μ*M ATP and a pre-determined calcium concentration, ranging from pCa 5 to pCa 9 was introduced. Free calcium concentrations and total ionic strength were determined using the binding constants and software program previously described^52^. The experimental buffer also contained a very low concentration of 1 *μ*m diameter silica beads (Bangs Inc.) coated with neutravidin, providing a linkage for attachment to the biotin/TRITC-labeled thin filaments and 100 nm excess Tn and Tm to ensure complete regulation of myosin binding to actin^53^. All experiments were performed at 30 C. Before data were collected, a control file was collected, where the dumbbell was close the surface but not able to interact with a myosin-coated 3 *μ*m diameter pedestal bead. This control trace was used to estimate trap stiffness by the equipartition method^54^. Typically several recordings of ~10 sec were obtained once binding events were observed over a pedestal. After these recordings were obtained the bead-actin-bead assembly was typically moved to another pedestal where more data records were obtained.

### Analysis of laser trap assay data

A custom program in Matlab was used to determine event frequency, duration, and displacement from the single molecule data, The algorithm, originally described by Page^55^, was previously adapted to analyze single molecule laser trap assay data^56^. This method uses changes in the variance of the signal by using probability density functions to detect individual actomyosin strong-binding events. In the present study, this method was employed to identify binding events. Once these events were identified, we determined the frequency of binding events during a given recording. The displacement and duration of each event was then determined by re-examining the raw displacement record. The onset of strong-binding was taken as the first positive value of the probability density function and the event termination was taken as the first negative value.

A separate custom Matlab program was used to analyze the mini-ensemble data, to determine peak force, event duration and the time between binding events for all recordings pCa5 to pCa7. This threshold based program was similar to the method we previously published^32^ with minor modifications, including automated detection. To be scored as a binding event, a displacement had to be greater than 8 nm and have lasted at least 10 ms. Imposition of this 10 ms threshold reduced the potential for the incorporation of “false” events, but also eliminated the ability to determine the frequency of very short duration events. This implies that we likely underestimated the total binding frequency in these mini-ensemble experiments. The threshold values where chosen based on (1) the lower limit of the average displacement for two-headed skeletal muscle myosin^57^ and (2) 10 ms being the minimum time threshold that, based on analysis of simulated data, resulted in no detection of false events. Once scored as an event, the algorithm would determine the peak force and duration for each event. We determined event frequency by dividing the number of events by the total time of the displacement recording, each of which lasted 10 s. Mini-ensemble data collected in the absence of Ca^++^ (pCa 9) were not amenable to either method of analysis described above. This was, in part, due to the shorter duration of binding events because of the elevated ATP concentration (100 *μ*M) and in part due to the low frequency of the binding events owing to the low Ca^++^ level. Therefore we took advantage of the well characterized drop in signal variance up myosin strong binding to actin^58^ to locate events and quantify the displacement/force and event duration. To do this a custom algorithm in Matlab located a 50% reduction in variance that lasted longer than 10 ms, using sliding window method. To validate our choice of variance threshold we analyzed recordings in which the bead-actin-bead assembly was held far from a myosin coated pedestal to determine if false events might be detected by our program. Using the same conditions and criteria we did not detect any binding events, suggesting that the analysis of the pCa 9 data did not include false events.

Determination of axial position of the thin filament in three-bead assay was performed to ensure that the height of the actin filament off the myosin coated pedestal was consistent across each Ca^++^ level in both the single molecule and mini-ensemble laser trap assay. Axial position of the thin was determined by analysis of the Airy disk diffraction pattern of the 3 *μ*m pedestal bead in brightfield images based on previously described methods^59, 60^. Brightfield images were taken either during or immediately after a data record to maintain the height of the actin filament above the myosin coated pedestal. Focusing above the 3 *μ*m pedestals keeps the image of them defocused. The more out of focus the 3 *μ*m pedestal bead the larger the radius of its central Airy disk^59^. We used this change in the Airy disk to determine to establish a linear relationship (*R* ^2^ = 0.991, see SM) between distance from the coverslip surface ranging from 2.5 *μ*m to 4.5 *μ*m above the surface. The initial calibration curve was developed by first bringing the surface into focus and then axially moving the piezo-controlled stage (Mad City Labs Inc.) in 1 nm increments in the *z*-axis, obtaining bright field images at each step. A custom script in MATLAB was developed to first determine the center of the disk. Experimental axial positions were determined by computing the Airy disk radius of the 3 *μ*m pedestal bead and then using the calibration curve to determine the height above the coverslip surface (see SM). Tests were performed with real height values determined using the same method described above, and the model was found to have a resolution of roughly 90 nm. The results suggested that the axial position of the actin filament ranged from 3.20 *μ*m to 3.45 *μ*m above the surface during data collection. This corresponds to a height slightly above the 3 *μ*m myosin coated pedestal. Importantly, there was no significant difference between the values for any condition, see SM), suggesting that any changes in frequency where due to the effects of Ca^++^ and not to a systemic difference in the distance between the actin filament and the myosin coated pedestal.

### Statistical analyses

Comparisons among the single molecule laser trap assay and motility data were determined using ANOVA and TukeyÕs post hoc tests to locate differences with the alpha level set at *p* < 0.05. The mini-ensemble data were not normally distributed therefore to compare the effects of Ca^++^ a non-parametric Kruskal-Wallis ANOVA and associated post hoc test was used to locate differences. These tests were performed using SigmaPlot 11.0.

## Electronic supplementary material

Supplementary Information
