# Supplementary material for: The molecular basis of thin filament activation: from single molecule to muscle
Source: Sci Rep. 2017 May 12;7:1822. doi: 10.1038/s41598-017-01604-8 (PMC5431769; doi:10.1038/s41598-017-01604-8)
Supplement: Supplementary file 1 — Supplementary Information [file 41598_2017_1604_MOESM1_ESM.pdf]

Supplementary material to accompany "The molecular basis of thin filament activation: from single molecule to muscle"

T. Longyear, S. Walcott and E. P. Debold

March 28, 2017

1 Supplementary experimental material

We performed a series of measurements of myosin's interaction with regulated thin filaments (RTFs, which consist of actin, troponin and tropomyosin). These experiments were performed at various calcium concentrations, with variable numbers of myosin, and under variable loading conditions. In some of these experiments, we measured event frequency. Here, as a supplement to the main text, we address the concern that this event frequency data might be affected by the height of the RTF above the flow cell surface. Additionally, in our model of these experiments (see section 2, Supplementary modeling material), we assume that Ca^{++} affects only myosin's attachment to a RTF. To support this assumption, in this supplement we also show that myosin's attachment duration and step size is Ca^{++}-independent. Finally, we show the results of a statistical analysis of our mini-ensemble data, showing that Ca^{++} has a significant effect on the maximum force and lifetime of binding interactions between myosin and actin observed in the laser trap.

1.1 Determination of thin filament height off the myosin coated pedestals

In the laser trap, event frequency is $\sim$ 20-fold slower than the weak-to-strong binding transition measured in solution ($40s^{-1}$ [1, 2]), because fluctuations in RTF height effectively reduce the concentration of actin. Additionally, the effective actin concentration is affected be the mean height of the RTF. In order to ensure that the height of the RTF off the myosin coated pedestal did not confound or influence the rate of myosin attachment to the thin filament in a manner independent of the [Ca^{++}], we determined the height of the RTF in three-bead assay in both the single molecule and mini-ensemble laser trap assay. The major concern was that the decreased frequency at lower [Ca^{++}] might have been due to the bead-RTF-bead assembly being further off the surface rather than to the decrease in Ca^{++}. Therefore axial position of the RTF was determined by analysis of the Airy disk diffraction pattern of the 3μm pedestal bead in brightfield images based on previously described methods [3].

Briefly, focusing above the 3μm pedestal beads generates a defocused image of the pedestal. The further the image is out of focus the larger the radius of its central Airy disk. This change in the Airy disk was used to establish the relationship between distance from the coverslip surface ranging from 2.5 to 4.5μm above the surface ($R^2 = 0.99$, Figure S1A). The initial calibration curve was developed by first bringing the surface into focus and then axially moving the 3-axis piezo-controlled stage (Nano-LPS 200, Mad City Labs, Inc.) in 1nm increments in the z-axis, obtaining still images at each increment. The fact that the determined height is slightly above 3μm likely reflects that the initial focal setting was based on a by-eye estimate. However since the major concern was the difference between Ca^{++} conditions, the absolute height determination was not important. Using the calibration to determine heights of objects with known values using the same method described above suggested a this method could detect a difference in height of at least 90nm.

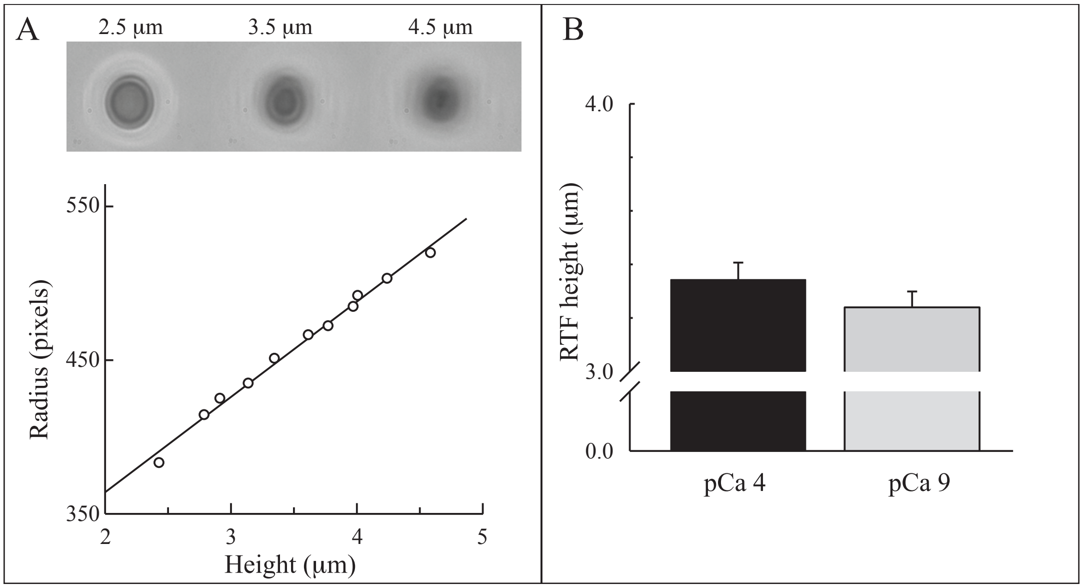

Figure S1: RTF height, which defines effective actin concentration, was constant for frequency measurements. A. Top, images of the 3μm pedestal bead at different heights above the surface based on movements of the piezo-controlled sub-stage. The radius of Airy disc of the bead was measured at various known heights and used to generated the calibration curve, shown below. B. The height during an experiment was determined from a still image taken during data collection where the height was determined based on the radius of the Airy disc using the calibration curve. The bar graph shows the average (mean ± SEM) thin filament height above the surface at pCa 4 and pCa 9 from the mini-ensemble laser trap assay. An ANOVA revealed no significant ($p < 0.05$) differences in filament height among all Ca^{++} levels in either the single molecule or mini-ensemble laser trap assays.

During a data collection in the laser trap assays, a brightfield image was obtained and the calibration curve was used to determine the corresponding height above the surface. The results suggested that the axial position of the RTF ranged from 3.20 to 3.45μm above the surface. This corresponds to a height slightly above the 3μm myosin coated pedestal. Importantly, there was no significant difference between the values for any condition. In fact there was a slight tendency to hold the filament closer to the pedestal at the lowest Ca^{++} level (pCa 9) compared to higher Ca^{++} (pCa 4) (Fig. S1B). Thus, any changes in frequency where due to the effects of Ca^{++} and not to a systemic difference in the distance between the RTF and the myosin coated pedestal.

Since RTF height did not vary systematically as a function of Ca^{++}, we can remove the effect of the reduced actin concentration in the single molecule laser trap measurements. In particular, given that actin concentration A is a function of average RTF height $\bar{z}$ the attachment rate of a single myosin molecule to a RTF measured in the laser trap is

$$k_{Trap} = \frac{A(\bar{z})}{K + A(\bar{z})} k_a(\mathrm{Ca}^{++})$$

where $k_a(\mathrm{Ca}^{++})$ is the attachment rate of myosin to a RTF at saturating actin. Similarly, the attachment rate of a single myosin molecule to an unregulated actin filament measured in the laser trap is

$$k^0_{Trap} = \frac{A(\bar{z})}{K + A(\bar{z})} k^0_a$$

where k^0_a is the attachment rate of myosin to an unregulated actin filament. The ratio of these rates is then

$$\frac{k_{Trap}}{k^0_{Trap}} = \frac{\frac{A(\bar{z})}{K+A(\bar{z})} k_a(\mathrm{Ca}^{++})}{\frac{A(\bar{z})}{K+A(\bar{z})} k^0_a} = \frac{k_a(\mathrm{Ca}^{++})}{k^0_a}$$

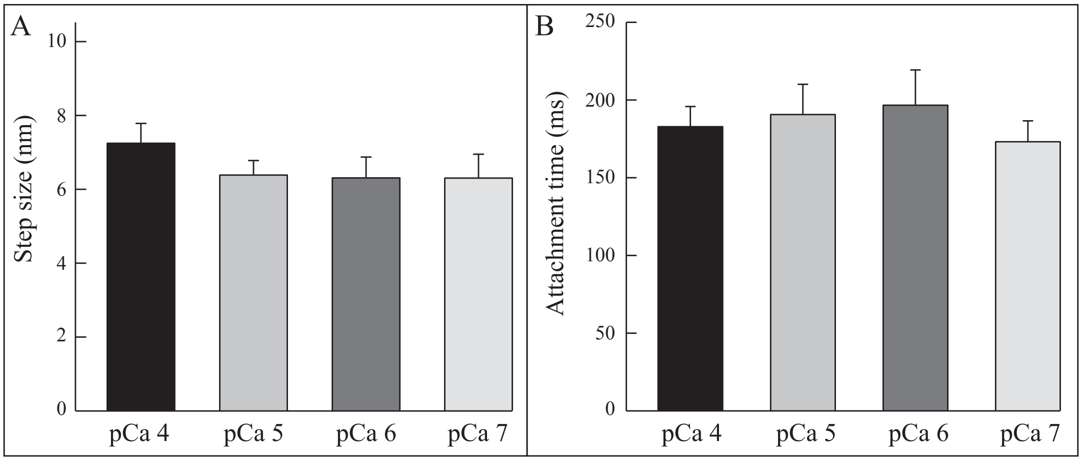

Figure S2: Single molecule step size and event durations do not depend on Ca^{++}. Values represent mean ± SEM. Data were collected at 1μM ATP. ANOVA revealed no significant differences among the Ca^{++} levels for either step size (A, $p = 0.46$) or event duration (B, $p = 0.79$). Sample size was 101, 99, 58, and 63 for pCa 4, 5, 6, and 7, respectively, with each observation representing a 1 to 3 minute recording of bead position.

Therefore, the ratio of attachment rates in the laser trap is the same as the ratio of attachment rates at saturating actin, the physiological condition. We define this ratio as ε.

1.2 Single molecule step size and binding event durations as a function of Ca^{++}

In addition to being used to determine the frequency of binding events (Fig. 1 Main text), the single molecule laser trap assay was used to determine myosin's single molecule mean step size and event duration at each [Ca^{++}], (Fig. S2). The step size and event durations were determined using the same method employed to determine the event frequency [4]. The mean step size was between 6 and 7 nm for all Ca^{++} levels. An ANOVA revealed no significant differences, suggesting that Ca^{++} did not affect myosins step size. The mean value of 6-7 nm is slightly lower than previous reports using whole skeletal muscle myosin binding to an unregulated actin filament [5]. However the value is nearly identical to the value previously observed when myosin binds to a RTF [6]. It was hypothesized that the decreased step size in the presence of regulation may be caused by TnTm affecting proper communication between the heads of myosin that is required for a 10 nm step [5, 6]. This prior work [6] was done in the absence of Ca^{++} therefore the present data suggest that step size is also reduced in the presence of saturating Ca^{++}. Event durations were also independent of the [Ca^{++}] (Fig S2). These data were collected at 1μM ATP and the duration is consistent with previous measurements with unregulated actin [6, 7].

In order to ensure that these measurements reflect the action of a single myosin molecule, the experiments were performed at a myosin concentration of 0.2 μg/mL. At this low concentration, we did not observe binding events at the majority of the pedestals on the flow cell surface. For example, the data collected at pCa 4 reflects interactions observed at 30 different pedestals with roughly 1 in 5 pedestals eliciting binding activity. The data at pCa 5 reflects 105 pedestals with the same ratio eliciting activity. At pCa 6 the data are from 50 pedestals with roughly 1 in 10 eliciting activity. Thus, at each different Ca^{++} concentration, our measurements reflect the binding frequency, step size and lifetime of at least five different single myosin molecules.

1.3 Statistics for mini-ensemble data

We performed a statistical analysis on our measurements of maximum event force and event lifetime from the mini-ensemble laser trap assay. The effect of [Ca^{++}] on peak force and event lifetime was determined

using an ANOVA using the data displayed in Fig. 3A of the main text. These data were not normally distributed ($p < 0.001$ Shapiro-Wilk test) therefore they were analyzed using the non-parametric Kruskal-Wallis ANOVA followed by a Dunns post-hoc test to locate significant differences. All pairwise comparisons were run based on ranks with the difference of ranks and critical value (Q) displayed, and whether this represented a significance difference ($p < 0.05$). The sample size was 615, 374, 223, 97 and 27 events for pCa 5, 6, 6.5, 7 and 9 respectively. The decrease in event number with decrease Ca^{++} reflects the Ca^{++} dependence of event frequency. For peak force all pairwise comparisons were significantly different except pCa 5 vs. 6 and pCa 6.5 vs 7 (Table S1). For event durations all pairwise comparisons were significantly different except pCa 5 vs. 6.5 (Table S2).

Comparison	Difference of ranks	Q	$p < 0.05$
pCa 5 vs pCa 9	717.235	9.454	Yes
pCa 5 vs pCa 7	317.498	7.533	Yes
pCa 5 vs pCa 6.5	194.698	6.456	Yes
pCa 5 vs pCa 6	23.996	0.948	No
pCa 6 vs pCa 9	741.231	9.641	Yes
pCa 6 vs pCa 7	341.494	7.768	Yes
pCa 6 vs pCa 6.5	218.693	6.700	Yes
pCa 6.5 vs pCa 9	522.537	6.647	Yes
pCa 6.5 vs pCa 7	122.800	2.617	No
pCa 7 vs pCa 9	399.737	4.762	Yes

Table S1: Statistics for maximum event force, mini ensemble data.

Comparison	Difference of ranks	Q	$p < 0.05$
pCa 5 vs pCa 9	643.067	8.477	Yes
pCa 5 vs pCa 7	270.896	6.427	Yes
pCa 5 vs pCa 6.5	47.807	1.585	No
pCa 5 vs pCa 6	142.941	5.650	Yes
pCa 6 vs pCa 9	500.125	6.505	Yes
pCa 6 vs pCa 7	127.955	2.911	Yes
pCa 6 vs pCa 6.5	95.134	2.914	Yes
pCa 6.5 vs pCa 9	595.259	7.572	Yes
pCa 6.5 vs pCa 7	224.089	4.754	Yes
pCa 7 vs pCa 9	372.170	4.433	Yes

Table S2: Statistics for event lifetime, mini ensemble data.

2 Supplementary modeling material

To understand and interpret our measurements, we used a model of myosin's interaction with a RTF [8, 9]. Here, as a supplement to the main text, we describe this model and demonstrate how it both shows that the data are self-consistent and also predicts how a myosin ensemble's interaction with a RTF changes with Ca^{++}.

2.1 Model description

The model contains two main components: the first component is a model for myosin's interaction with actin and associated ATP hydrolysis [7]; the second component is a model for tropomyosin-induced coupling between nearby myosin molecules [8, 9, 10, 11].

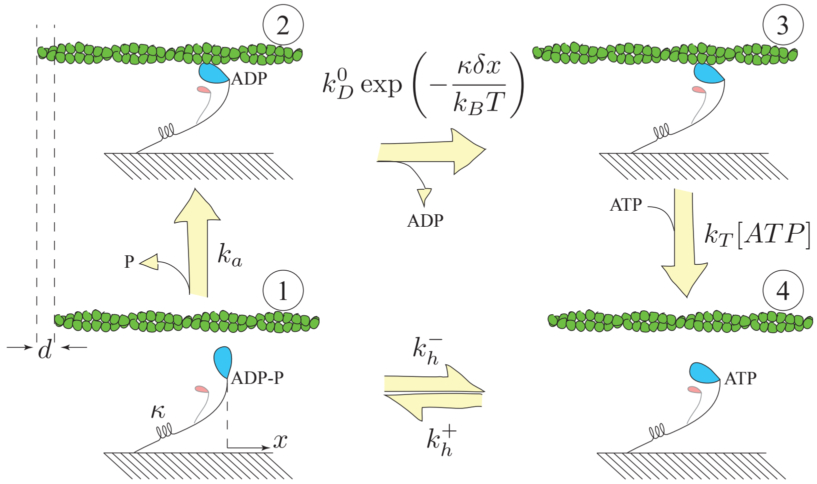

Figure S3: Cartoon of myosin's ATP-dependent interaction with actin. Starting at the lower left (state 1), myosin (in blue) binds to actin (green), releases phosphate and undergoes a power stroke, though not necessarily in that order. Completion of these steps moves an unloaded actin filament a distance d and puts myosin in the strongly bound state (state 2). Myosin then releases ADP in a force dependent process, where force is the extension of myosin x times its linear stiffness κ, transitioning to the rigor state (state 3). Upon ATP binding, myosin rapidly unbinds from actin (state 4). The reversible hydrolysis of ATP completes the cycle.

2.1.1 Myosin's ATP-dependent interaction with actin

The model for myosin's interaction with actin and hydrolysis of ATP contains four distinct kinetic states. They are (as numbered in Fig. S3) (1) an unbound/weakly bound state, where myosin's "head" is in a pre-powerstroke position and has hydrolyzed ATP (ADP and inorganic phosphate, P) in its active site; (2) a strongly bound state, where myosin's head is in a post-powerstroke position and has ADP in its active site; (3) a rigor state, where myosin's head is in a post-powerstroke position and its active site is empty; and (4) an unbound/weakly bound state, where myosin's head is in a post-powerstroke position and has ATP in its active site.

Each transition between states is associated with a change in conformation and/or a change in the nucleotide state of the myosin head. In the transition from (1) to (2), P is released and the powerstroke occurs (though not necessarily in that order). Since this state transition is also associated with the myosin head binding to actin, and since it binds in the pre-powerstroke position, if the actin filament's movement is unimpeded this results in a movement of the actin filament of a distance d. In the transition from (2) to (3), ADP is released. ATP binds and the myosin head detaches from actin in the transition from (3) to (4). Finally, in the transition from (4) back to (1), ATP is hydrolyzed and the powerstroke is reversed. Note that, in the absence of P and ADP in solution, each transition is assumed irreversible, except the transition from (4) to (1).

In either of the bound states (states 2 and 3), myosin can experience force, either from an external influence (like the laser trap) or from other myosin molecules interacting with the same actin filament. These forces affect only one reaction rate, the transition from state (2) to (3) associated with ADP release [12, 13]. Because ADP release rate depends on force, myosin molecules are mechanochemically coupled, in the sense that a myosin molecule that binds and then undergoes its powerstroke (i.e. transitions from state

(1) to (2)) applies forces to other myosin molecules that have previously attached to the actin filament. These forces then influence myosin's chemistry, by changing the transition rate from state (2) to (3). Generally, it is most convenient to keep track of the extension of each myosin molecule (x), and then relate that to force by assuming myosin behaves as a linear spring of stiffness κ [14].

Parameter	Value without regulation (Walcott et al. 2012)	Value with regulation
k_h^+	100 s^{-1}	100 s^{-1}
k_h^-	10 s^{-1}	10 s^{-1}
k_a	40 s^{-1}	40 s^{-1}
k_D^0	350 s^{-1}	350 s^{-1}
k_T	2 $\mu M^{-1} s^{-1}$	3 $\mu M^{-1} s^{-1}$
d	10 nm	10 nm
δ	1.86 nm	1.86 nm
κ	0.3 pN/nm	0.3 pN/nm

Table S3: Parameters for the model shown in Fig. S3.

This model describes a wide range of molecular-scale measurements performed with chicken pectoralis myosin at ~ 25– $30°$C, the same muscle type and temperatures used in our experiments [7, 15]. Importantly, the model describes experiments with single molecules in the laser trap [7], the distribution of maximum forces and lifetimes for myosin binding events with mini molecular ensembles in the laser trap [15], and the speed of actin filaments in the motility assay as a function of ATP ([7]. These are the three experiments performed here, but with RTFs at variable Ca^{++} as opposed to unregulated actin filaments. That the model can describe each experiment with the same set of parameters (listed in Table S3) suggests that it adequately characterizes the interaction of myosin with actin; thus, modeling our current experiments only requires a description of the effects of Ca^{++} on the attachment of, and the local coupling between, myosin molecules. While it is possible that the individual parameters are inexact – for example, the parameter $\delta = 1.86$ nm is in broad agreement with, but larger than, some measurements (Capitanio et al. 2006 [16] predict an upper bound of 1–1.3 nm for this value, and Greenberg et al. 2014 [17] and Sung et al. 2015 [18] measure $\delta = 0.96$ and 1.01 nm, respectively, for cardiac myosin) – at the very least the model captures the emergent behavior that occurs when an ensemble of myosin work together.

While there was no need to adjust the majority of the parameters in this kinetic model, our measurements from the motility assay suggest an ATP binding rate of $k_T = 2.95 \pm 0.45 \mu M^{-1} s^{-1}$ (mean plus/minus SD, see Fig. S4). In our previous work [7], we estimated an ATP binding rate of $k_T = 2 \mu M^{-1} s^{-1}$. It is unclear whether this change in ATP binding rate is due to the presence of regulatory proteins, or simply experimental variability. Thus, in our simulations, we use an ATP binding rate of $k_T = 3 \mu M^{-1} s^{-1}$, while all other parameters remain the same (see Table S3).

2.1.2 Modeling Ca^{++}-dependent activation

Myosin molecules, interacting with a RTF, influence the position of Tm, which wraps around actin. Our model belongs to a class of models of Ca^{++}-dependent activation that derive equations relating these mechanical interactions into chemical binding rates. As a starting point, we use a model that simplifies the geometry and mechanics of the system, the continuous flexible chain model [19, 20, 21]. The basic idea of this model is to treat Tm as an infinite, slender, linear elastic beam. The interaction of Tm with actin and Tn is treated as a potential energy density $W(x)$ that, for simplicity, is assumed not to vary along actin. When myosin binds to actin, it induces a displacement in Tm, and the slope of the beam is locally zero. Thus, mathematically, the position of Tm ($u(x)$) is given by the solution to the following equation

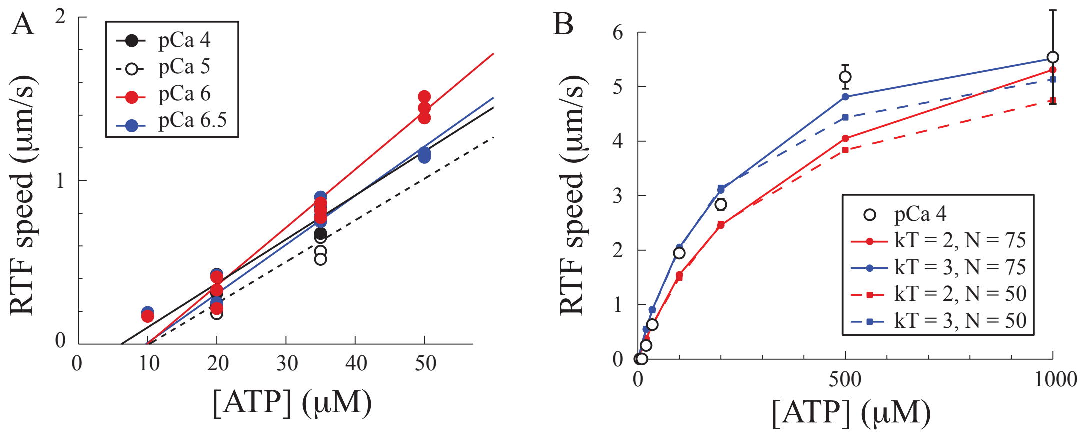

Figure S4: Motility data with RTFs at saturating Ca^{++} are consistent with a higher ATP binding rate and larger ensemble size than motility data with actin alone. A. The initial slopes of our motility data suggest an ATP binding rate of $k_T = 2.95 \pm 0.45 \mu M^{-1}s^{-1}$ (mean plus/minus SD). At low ATP concentrations, RTF speed (v) is limited by ATP binding so that $v = dk_T[ATP]$, so the initial slope, divided by the powerstroke size, d, gives k_T. This result motivates our choice of $k_T = 3\mu M^{-1}s^{-1}$ in our simulations. B. Comparison of simulations to our motility data support $k_T = 3\mu M^{-1}s^{-1}$ and an ensemble size, $N_{mot} = 75$. With $N_{mot} = 50$, estimated from motility with actin alone (Walcott et al. 2012), increasing k_T from 2 to $3\mu M^{-1}s^{-1}$ gives better agreement with our measurements, but cannot match the RTF speeds observed at high [ATP]. Increasing ensemble size to $N_{mot} = 75$ allows the model to recreate these speeds.

for a beam on a (potentially non-linear) elastic foundation:

$$EI\frac{d^4u}{dx^4} + \frac{dW}{dx} = 0 \tag{1}$$

where E and I are the Young's modulus and moment of inertia of the Tm beam, respectively. To find the position of Tm between two bound myosin, separated by a distance s and each inducing a displacement D, one would solve this equation subject to the boundary conditions that $u(0) = u(s) = D$, and $u'(0) = u'(s) = 0$ (where prime indicates differentiation with respect to x).

The solution to Eq. 1 only gives the lowest energy conformation of the beam. However, being a molecular scale system, Tm is constantly sampling other energy conformations. Calculating, say, the equilibrium position of the beam therefore requires an estimate of the energy of all beam conformations – a complex problem that requires further assumptions about precisely how myosin and Tm interact. For example, Smith 2001 [19] assumes that myosin locally "pins" Tm in place. However, the precise nature of this myosin-Tm interaction is unknown.

The problem is simplified considerably with a different assumption: rather than constraining Tm locally, it is assumed that myosin binding has little effect on the distribution of energies available to Tm. Then, myosin does not effect the entropy of Tm, and the free energy change upon myosin binding depends only on the lowest energy conformation of the beam – a quantity that follows simply from the solution to Eq. 1. Furthermore, since we are only interested in the lowest energy conformation of the beam, further simplifications are possible.

There are two main contributions to the energy of the Tm beam. First, there is an elastic energy cost to deforming the Tm beam. Second, there is an interaction energy between the Tm beam and actin/Tn, defined by $W(u)$ integrated along actin. Suppose that we wish to calculate the energy of the Tm beam between two bound myosin motors, separated by a distance s. Then, the interaction energy is given by $\int_0^s W(u)dx$. If s is small, this energy cost is small, and elastic energy dominates. Thus, if two nearby myosin molecules are bound, the intervening Tm beam is (approximately) straight, minimizing the elastic energy. Then the energy of the beam, U, should be $U \approx \int_0^s W(D)dx = W(D)s$, increasing linearly with the

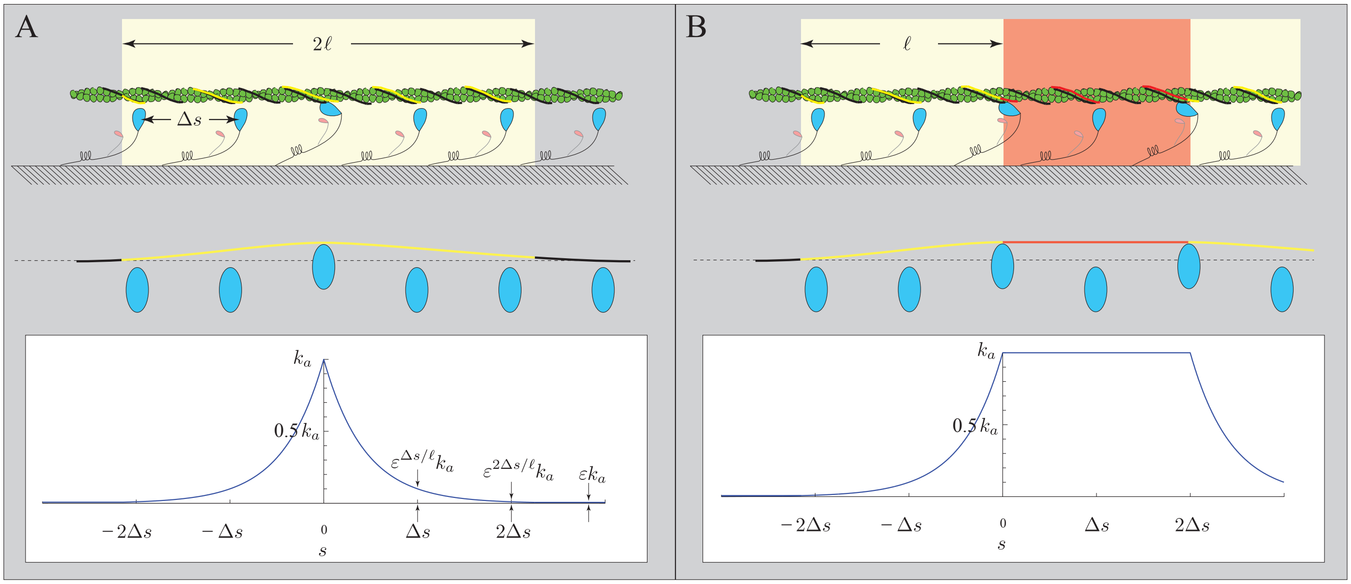

Figure S5: The regulation model, including a definition of the parameters ℓ and ε. Myosin is in blue, actin in green, tropomyosin in black [inactive], yellow [partially active] and fully active [red], troponin is not shown. In each plot, top shows a cartoon of muscle; middle shows the simplified physical model with myosin heads inducing a fixed displacement to tropomyosin, which is modeled as a slender, infinite, linear-elastic beam; and bottom shows the attachment rate as a function of position, calculated from Eq. 2. A. When the first myosin binds, it partially activates the RTF filament over a distance ℓ to its left and right. B. If a subsequent myosin molecule binds, within ℓ of the first one, it fully activates the RTF.

separation between the bound myosin. Alternatively, is s is large, the interaction energy cost dominates, and the beam minimizes this value. The interaction energy is minimized when each myosin induces an independent deformation in Tm, giving an energy cost $U \approx U_0$, independent of s. Thus, there are two regimes, an initial linear rise in energy and a subsequent plateau. We can further simplify the problem by assuming that the transition between these two regimes is abrupt [8].

This energy function, $U(s)$, is then defined by two parameters. The first, we define as $\varepsilon = \exp(-U_0/k_BT)$, which is dimensionless; the second, we define as $\ell = U_0/W(D)$ and has units of length. It is often convenient to define the non-dimensional parameter $\mathcal{C} = \ell/\Delta s$, scaling this distance by the spacing between molecules Δs [8, 9, 10, 11], but since Δs varies in our experiments, here we prefer to use the dimensional parameter ℓ. Note that both of these parameters are defined in terms of the molecular mechanics of Tm, i.e. given E, I and the interaction energy function $W(x)$, we could calculate both ε and ℓ. With a few additional assumptions, these two parameters also define how Tm induces local coupling between myosin molecules. We now discuss these assumptions.

In the kinetic model (Fig. S3), the transition from state (1) to state (2), called the weak-to-strong binding transition, contains a series of sub-steps. Given that one of these substeps is a rapid equilibrium between the weak/unbound states and that the equilibrium favors the unbound state e.g. [22], the net attachment rate is

$$k \approx \frac{K_w}{1+K_w} k_{ws} \approx K_w k_{ws} \approx k^0 \exp\left(-\frac{U}{k_B T}\right)$$

Thus, supposing that a given molecule's i^{th} neighbor to the left and j^{th} neighbor to the right are bound

to actin, then that given molecule has an attachment rate k from the following equation [9, 11].

$$k(i,j,\mathcal{C},\varepsilon) = \begin{cases} \varepsilon k^0 & : \; i,j \geq \mathcal{C} \\ \varepsilon^{i/\mathcal{C}} k^0 & : \; i < \mathcal{C}, j \geq \mathcal{C} \\ \varepsilon^{j/\mathcal{C}} k^0 & : \; i \geq \mathcal{C}, j < \mathcal{C} \\ \varepsilon^{(i+j-\mathcal{C})/\mathcal{C}} k^0 & : \; i,j < \mathcal{C}, i+j > \mathcal{C} \\ k^0 & : \; i+j \leq \mathcal{C} \end{cases} \tag{2}$$

where $\mathcal{C} = \ell/\Delta s$. As this attachment rate depends on the state of nearby molecules, it introduces local coupling to the model.

This attachment rate, which models the effect of Tm, is defined by the two parameters, ε and ℓ. Both parameters depend on $W(x)$, the interaction of Tm with actin and Tn, the latter of which changes state depending on Ca^{++}, and so both are potentially functions of Ca^{++}. Lacking details of the Ca^{++}-dependence of $W(x)$, we again make simplifying assumptions.

The first simplifying assumption is that ℓ does not depend on Ca^{++}. A value of $\ell = 400$ nm has been previously measured, based on fits to in vitro motility [8, 10], and also based on direct measurements of myosin molecules binding to a RTF in the laser trap [6]. Thus, here we fix $\ell = 400$ nm, regardless of Ca^{++}.

Note that ℓ defines how far partial activation spreads upon the binding of one myosin (Fig. S5), and is not equal to a "cooperative unit" which is the distance that full activation spreads upon the binding of one myosin [23]. Although the relationship between the two varies depending on experiment, a value of $\ell = 400$ nm predicts a transition from single molecule binding events to motility in the laser trap at a spacing of 107 nm, which compares very well to direct measurements of this value of 111 nm [6, 8].

The next simplifying assumption is that the binding of Ca^{++} to Tn is fast. Then, supposing that the energy required for myosin to bind to a RTF in the absence of Ca^{++} is ΔG_B, and that energy is ΔG_C when Tn is saturated with Ca^{++},

$$\varepsilon = \exp(-\theta \Delta G_C - (1-\theta)\Delta G_B) = \varepsilon_{min}^{1-\theta} \varepsilon_{max}^{\theta} \tag{3}$$

where θ is the proportion of Tn saturated with Ca^{++}, and defining $\varepsilon_{min} = \exp(-\Delta G_B)$ and $\varepsilon_{max} = \exp(-\Delta G_C)$, where ΔG is measured in units of Boltzmann's constant times absolute temperature. Finally, we assume Michaelis-Menten saturation of Tn with Ca^{++}

$$\theta = \frac{[Ca^{++}]}{K + [Ca^{++}]} \tag{4}$$

which may be an oversimplification, because there are two main Ca^{++} binding sites on Tn. We examine this assumption later in this supplement (section 2.4.4, Michaelis-Menten binding of Ca^{++} to Tn).

With these assumptions, the model reasonably describes fiber-level experiments, including isometric force as a function of Ca^{++} and twitch summation [9]. Additionally, direct measurements of fluorescent myosin binding to RTFs in solution provide an estimate of $\varepsilon_{max} = 0.5$ [11], and measurements of in vitro motility ($\varepsilon_{min} = 0.0030$ [8]), single molecule binding frequency ($\varepsilon_{min} = 0.0045$ [6]) and fluorescent myosin binding to RTFs ($\varepsilon_{min} = 0.010$ [11]) give an estimate of $\varepsilon_{min} = 0.006$ (the mean of the three). Given these assumptions, Ca^{++}-dependent regulation is completely specified by a single free parameter, K.

2.2 Parameter estimation

The activation model is defined by the single parameter, K. This parameter defines how Ca^{++} affects myosin's attachment to a RTF via Eqs. 3 and 4. Here, we discuss how we estimated K from our measurements in each of three experimental systems.

2.2.1 Single molecule laser trap

In the single molecule laser trap, we directly measure the rate that a single myosin molecule binds to a RTF. Since the single molecule has no neighboring myosin, Eq. 2 gives an attachment rate of εk^0. By dividing this frequency by the single molecule binding frequency we measured in the absence of regulation ($k^0 = 2.2\text{s}^{-1}$), this is a direct measurement of ε.

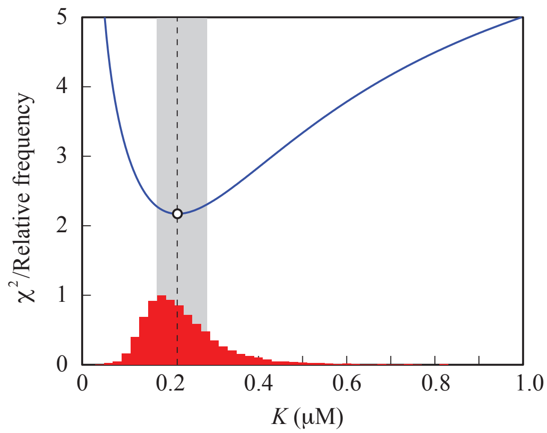

Figure S6: Estimating K from single molecule fits. Varying K and comparing Eq. 3 and 4 to the data gives a curve of χ^2 as a function of K. The minimum of this curve, $K = 0.216\mu\text{M}$, is the best-fit and is not significantly different from the data ($p > 0.05$, χ^2 test). Using a bootstrapping, we can determine the uncertainty in this estimate. A histogram of best fit K values is shown in red. This distribution is strongly non-normal, with a long tail. The median of this distribution, $K = 0.215\mu\text{M}$, is nearly identical to the best-fit value of K and the lower and upper quartiles are $K = 0.170\mu\text{M}$ and $K = 0.279\mu\text{M}$, respectively. These give an uncertainty estimate of $\pm 0.055\mu\text{M}$, the shaded region.

We calculated the best fit by minimizing the χ^2 error, which gives $K = 0.216\mu\text{M}$. The minimum χ^2 value is 2.17, well below the critical χ^2 value (at which $p = 0.05$) of 9.488. To estimate the error associated with this value, we used a bootstrapping approach. At each pCa concentration, we randomly selected from our N frequency measurements, with replacement, until we had a full complement of N measurements. We then fit the resulting data, using Matlab's fminsearch function (a Nelder-Mead simplex algorithm) with an initial random seed to find the value of K that minimizes mean squared error. We repeated this process 10,000 times, giving a distribution of best-fit K values. The distribution is strongly non-normal, with a long tail at high K (see Fig. S6). However, the median of this distribution was $K = 0.215\mu\text{M}$, nearly identical to the best-fit value of K. The lower quartile was $K = 0.170\mu\text{M}$ and the upper quartile was $K = 0.279\mu\text{M}$. We report the best-fit plus/minus half the difference between the upper and lower quartile, $K = 0.216 \pm 0.055\mu\text{M}$. Note that similar results are obtained regardless of whether we minimize χ^2 error or average mean squared error.

2.2.2 Mini-ensemble laser trap

Estimating K from the mini-ensemble laser trap measurements is complicated for two reasons. First, we must estimate two parameters, N_M and k_a^2, defined below; and second, simulations of a single experiment are computationally expensive, requiring several minutes to run. In the following, we (1) describe the model of these experiments, defining the parameters N_M and k_a^2 in the process; (2) explain how N_M and k_a^2 were estimated; and (3) explain how K was estimated. In addition to these parameters, we also had to estimate the degree of coupling, i.e. the value of $\ell/\Delta s$ in Eq. 2. We used $\ell/\Delta s = 3$, meaning that each myosin molecule is coupled to its three closest neighbors on either side. A justification for this estimate is given in section 2.3.1 of this supplement.

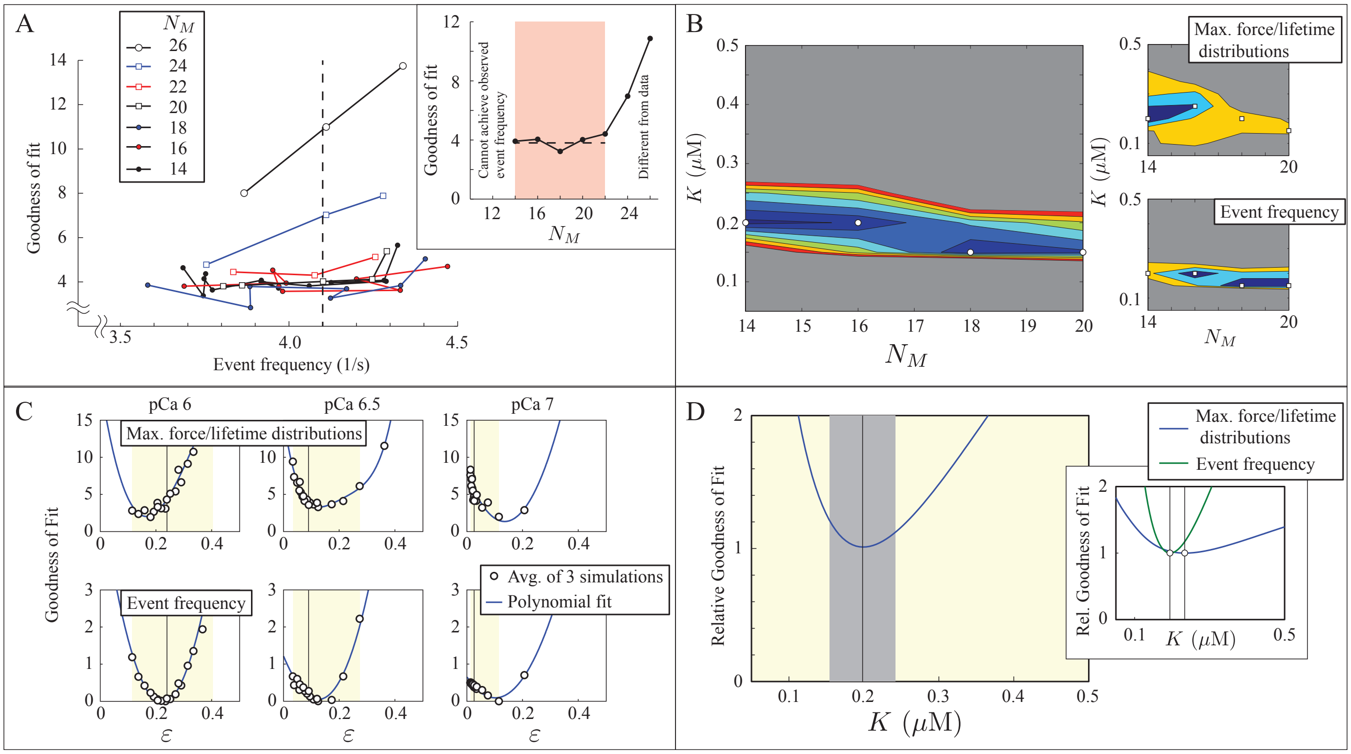

Figure S7: Estimating N_M, k_a^2 and K from mini-ensemble fits. A. Curves of goodness of fit as a function of event frequency for several values of N_M. Event frequency was varied by changing k_a^2. At the measured event frequency (dashed line) goodness of fit was determined, giving goodness of fit as a function of N_M (inset). Below $N_M = 14$, the observed event frequency could not be achieved without increasing k_a^2 above k_a; above $N_M = 22$ the fit dramatically worsens. B. The best-fit value of K is insensitive to N_M. A contour plot, showing goodness of fit (cold colors indicate a better fit; gray area is a worse fit than the dark red) as a function of K and N_M. Insets show individual fits to the maximum force/lifetime distributions (top) and event frequency (bottom). In all cases, the best fit $K \approx 0.2\mu$M. C. A series of simulations at $N_M = 14$, showing goodness of fit as a function of ε at increasing pCa (left to right). Top shows fits to maximum force/lifetime data; bottom shows fits to event frequency data. To minimize the effect of noise, each point represents the average of three simulations, and we fit the simulated data with a polynomial (sold line). Yellow region shows the plausible range of K (from 0.05 to 0.5μM), and the vertical line indicates the best fit, $K = 0.199\mu$M. D. Goodness of fit as a function of K. Best fit (vertical line) plus/minus one standard deviation (shaded region) is shown. Inset shows individual fits to maximum force/lifetime distributions and event frequency, which are weighted equally in the main plot.

The model

To model these small myosin ensembles, we used our previous approach for unregulated actin [15], with some modifications. Briefly, this method involves using a modified version of the Gillespie algorithm [24] to keep track of the state of each myosin molecule in a small ensemble as they interact with a common actin filament. The filament moves, due to Brownian motion, and when a myosin molecule binds to it, the mean position of the actin filament (and the laser-trapped beads) moves and motion is restricted.

In our previous work [15], we assumed that the motion of the bead was well-approximated by white noise from a Gaussian distribution with mean position given from mechanical equilibrium of the myosin-actin-bead-laser system, and standard deviation $\sigma = \sqrt{k_B T/k}$ where k is the overall stiffness of the myosin-actin-bead-laser system. This overall stiffness includes the trap stiffness (which we measured for each individual data recording) and each bound myosin molecule. Here, in order to model the noise more accurately, we include a time scale for the noise. In particular, we model the motion of the bead-actin-bead system as an over-damped system with a damping coefficient of $\gamma = 2.25 \cdot 10^{-5}$pN $\cdot$ s/nm. We then use

a simple forward Euler integration scheme to integrate the stochastic Langevin equation, and update the position of the bead-actin-bead system at each time interval (0.0025 s). The damping coefficient, γ, was picked to give a signal with a similar power-spectrum to our measurements.

We found in our previous work [15] that mini myosin ensembles exhibit a form of binding cooperativity. Specifically, the first myosin molecule binds relatively slowly (2.2s^{-1}), the second myosin molecule at a faster rate, and subsequent molecules at a rate that approaches the weak-to-strong binding transition measured in solution (40s^{-1} [1, 2]). The likely physical basis of this cooperativity is fluctuations in the height of the bead-actin-bead system that are restricted upon myosin binding. To model this effect, we assumed that the second myosin molecule bound at a rate, k_a^2, that was somewhere between the single molecule and solution rate.

Estimating N_M and k_a^2

Besides this unknown binding rate (k_a^2), we also do not precisely know the number of myosin molecules in the mini-ensemble (N_M). We estimated these values by fitting the data collected at near-saturating Ca^{++}, pCa 5.

To fit the data, we first simulated the laser trap/mini ensemble system. These data were analyzed with the same algorithm used to analyze the experimental measurements, until we had the same number of binding events. At that point, we compared the distribution of event lifetimes and maximum forces, by calculating the mean-squared difference between the measured and simulated cumulative probability distributions. We then determined the event frequency in our simulations by dividing the number of events by the total time. We performed these simulations for ensemble sizes of $N_M = 14, 16, 18, 20, 22, 24$, and 26 (see Fig. S7A). We found that ensemble sizes of $N_M = 13$ or fewer were unable to achieve the observed event frequency with k_a^2 less then the solution weak-to-strong binding transition rate of 40s^{-1}. For the remaining larger ensembles, we varied k_a^2 to get event frequencies that were both lower and higher than the observed frequency (4.1s^{-1}) and interpolated to get the predicted goodness-of-fit at the observed event frequency. For each value of N_M and k_a^2, we performed three simulations and found the average event frequency and goodness-of-fit of the three.

These simulations show that there is little difference in goodness-of-fit for ensemble sizes less than 22, but that ensemble sizes of 24 and 26 are different from the data (Fig. S7A, inset). Thus, we conclude that ensemble size, N_M, is between 14 and 22. For each N_M, there is an associated best-fit k_a^2 based on the binding frequency ($k_a^2 = 10, 13, 17, 25$ and 36s^{-1} for $N_M = 22, 20, 18, 16, 14$, respectively). In general, these values are higher than our previous estimate of $k_a^2 = 1 + 2\text{s}^{-1}$. One reason for this difference could be that RTFs are stiffer than actin alone [25]. Thus, the binding of the first myosin molecule would restrict the motion of an RTF more than an actin filament, which might flex and thereby inhibit the binding of a second myosin. Another possibility is that, in this study, we have event frequency information, which was lacking in our previous study, which makes the current estimate more accurate.

Estimating K

We determined K by fitting the remaining measurements, at pCa 6, 6.5, and 7. Note that, although we collected data at pCa 9, we used a different event detection algorithm to analyze these data, so we did not simulate them.

As before, to fit the data, we first simulated the laser trap/mini ensemble system and then analyzed the data with the same algorithm used to analyze the experimental measurements, until we had the same number of binding events. Data and model were compared by calculating the mean-squared difference between the measured and simulated cumulative probability distributions of event lifetimes and maximum forces. Event frequency in our simulations was determined by dividing the number of events by the total time, and compared to measurements by determining the sum of the squared difference of the mean

frequencies. We looked at ensemble sizes of $N_M = 14, 16, 18, 20$ – all values that were consistent with the pCa 5 data, and using the best-fit values of k_a^2 ($k_a^2 = 36, 22, 20, 17$, and 13, respectively). We then varied K from $0.05, 0.1, 0.15, 0.2, 0.25, 0.3$, and 0.35μM. At each K, N_M combination, we performed three simulations and found the average event frequency and goodness-of-fit of the three (a total of 90 simulations of the experiments at the three pCa values).

Interestingly, there is little effect of N_M on the best-fit value of K, either for the fits to event frequency ($K = 0.002, 0.002, 0.0015, 0.0015$, for $N_M = 14, 16, 18, 20$, respectively) or the fits to the distribution of event lifetimes and maximum force ($K = 0.002, 0.0025, 0.002, 0.0015$, for $N_M = 14, 16, 18, 20$, respectively). When combined, with equal weight, the range of viable values of K fell between 0.14 and 0.27μM, regardless of N_M (Fig. S7B).

To get a more precise estimate of K, we performed a larger set of simulations at $N_M = 14$, which is most consistent with our previous estimate of ensemble size $N_M = 21 \pm 3$, given that we used 10μg/mL here and 15μg/mL in our previous measurements. To minimize the effect of stochastic noise, we fit the resulting goodness-of-fit curves with polynomials (Fig. S7C). Then, the optimal value of K was 0.195μM for event frequency and 0.235μM for the distributions of event lifetimes and maximum force (Fig. S7D, inset). Given the two fits equal weight gives a best-fit estimate of $K = 0.199\mu$M (Fig. S7D). Since the simulations are so computationally expensive, a bootstrapping approach for an uncertainty estimate was impractical. Instead, assuming that the model with the best-fit value of K was exact, we estimated the standard deviation from each measurement (distribution of event lifetimes, distribution of event maximum forces and event frequency). Then, we calculated the χ^2 error, and found the values of K at which $p = 0.05$, thereby identifying our 95% confidence interval. The standard deviation was estimated as one quarter of this interval, giving $K = 0.199 \pm 0.044\mu$M.

In the main text (Fig. 3a), we present the distributions of event lifetimes and event maximum forces both for the model and the measurements. As an alternate view of the data, here we present average event lifetimes and maximum forces (Fig. S8). Generally, we observe that lifetimes and forces increase with Ca^{++}. The slight decrease in maximum force at pCa 5 is explained by differences in trap stiffness, since maximum event displacement increases monotonically with Ca^{++} (Fig. S8, inset).

2.2.3 In vitro motility

Estimating K from the in vitro motility measurements required an estimate of one parameter, N_{mot}, the number of myosin molecules interacting with an RTF. In the following, we (1) describe the model of motility; (2) explain how N_{mot} was estimated; and (3) explain how K was estimated. In addition to these parameters, we also had to estimate the degree of coupling, i.e. the value of $\ell/\Delta s$ in Eq. 2. We used $\ell/\Delta s = 11$, meaning that each myosin molecule is coupled to its eleven closest neighbors on either side. A justification for this estimate is given in section 2.3.1 of this supplement.

The model

We have previously modeled in vitro motility with RTFs [8, 10]. We used the same approach. As with the mini ensemble simulations, we used a modified version of the Gillespie algorithm [24] to keep track of the state of each myosin molecule in a small ensemble as they interact with a common actin filament. The filament moves due to Brownian Motion and when a myosin molecule binds to it. The motion is determined by calculating the position at which all forces balance; that is, we assume that the myosin-RTF system reaches mechanical equilibrium instantly. To determine filament velocity, we simulated 30,000 chemical reactions (myosin binding, ADP release, ATP binding/detachment, and ATP hydrolysis), fit the latter half of the position-time trace with a straight line (we neglect the first half to allow the system to reach steady state), and filament velocity was the slope of that line.

As shown in Fig. S4B, our previous estimate of $N_{mot} = 50$ underestimated RTF speed. Increasing the

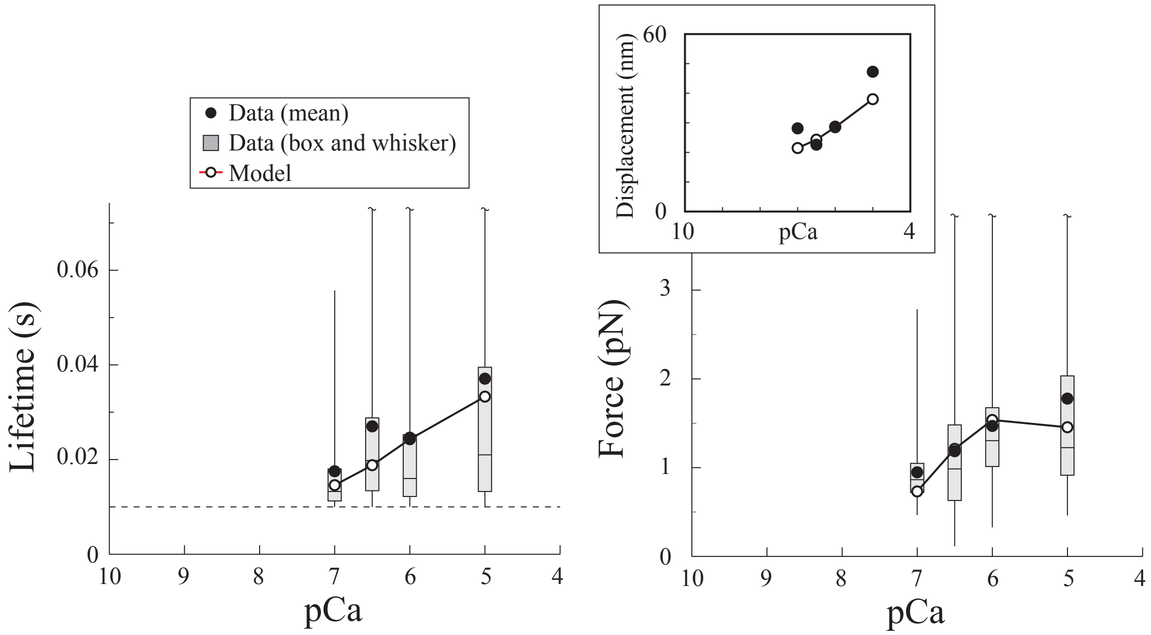

Figure S8: The model reproduces the Ca^{++}-dependence of the average maximum force and lifetime of binding events measured using a laser trap with a mini myosin ensemble. Left, average event lifetime increases monotonically with Ca^{++}. The dashed line indicates the minimum event lifetime of our event detection algorithm (10 ms). Right, average maximum event force generally increases with Ca^{++}, although pCa 5 shows a slight decrease with respect to pCa 6 for the model and for the median measured force. This non-monotonicity is likely due to differences in trap stiffness, since it disappears when maximum event displacement is plotted (inset). Note, for all data at pCa < 7, the upper limit of the whisker extends beyond the axis limits.

ensemble size to $N_{mot} = 75$ gave better agreement with the data. One potential reason for this larger value is that RTFs are stiffer than actin [25]. In the model, the RTF is assumed to be infinitely stiff. Thus, if a myosin molecule binds and undergoes its powerstroke, it applies forces to all bound myosin. In reality, if myosin molecules are too distant (i.e. over a persistence length away), the newly bound molecule will not apply a force on them. Thus, the parameter N_{mot} gives an estimate for the number of myosin molecules that are mechanochemically coupled. The stiffer the filament, the larger N_{mot} should be.

Estimating K

To estimate K, at each pCa we fit the velocity-ATP curve by varying ε. To do so, we simulated 100 velocity-ATP curves with ε at equally spaced intervals between 0 and 0.5 (i.e. $\varepsilon = 0.005, 0.010, 0.015, \ldots, 0.495, 0.500$). We then used a Bootstrapping method to estimate the distribution of best-fit ε values. Specifically, we randomly selected velocity data points, with replacement, at each ATP concentration and determined the best-fit ε. Performing 10,000 of these fits gave estimates of $\varepsilon = 0.491 \pm 0.012$ (pCa 4), $\varepsilon = 0.486 \pm 0.017$ (pCa 5), $\varepsilon = 0.236 \pm 0.079$ (pCa 6), $\varepsilon = 0.139 \pm 0.051$ (pCa 6.5), $\varepsilon = 0.025 \pm 0.002$ (pCa 7) and $\varepsilon = 0.008 \pm 0.001$ (pCa 9) (mean plus/minus SD, see Fig. S9A). Note that, to obtain a more precise estimate of ε at pCa 9, we re-ran simulations with 10 values of ε equally spaced between 0 and 0.01. These estimates are independent of our assumed form of ε, given by Eqs. 3 and 4.

Fitting the resulting $\varepsilon(Ca^{++})$ curve with Eqs. 3 and 4 gives an estimate of $K = 0.217 \pm 0.024 \mu M$ (best fit not significantly different from data, $p > 0.05$, χ^2 test; standard dev. estimated by one quarter of the range of K that are not sig. diff. from the data). For most pCa values, the fits using the best fit ε and the fits using Eqs. 3 and 4 with $K = 0.217\mu$M are nearly indistinguishable (Fig. S9B). At pCa 6.5, there is a difference between the two, but the data also show a large discrepancy at the highest ATP concentration

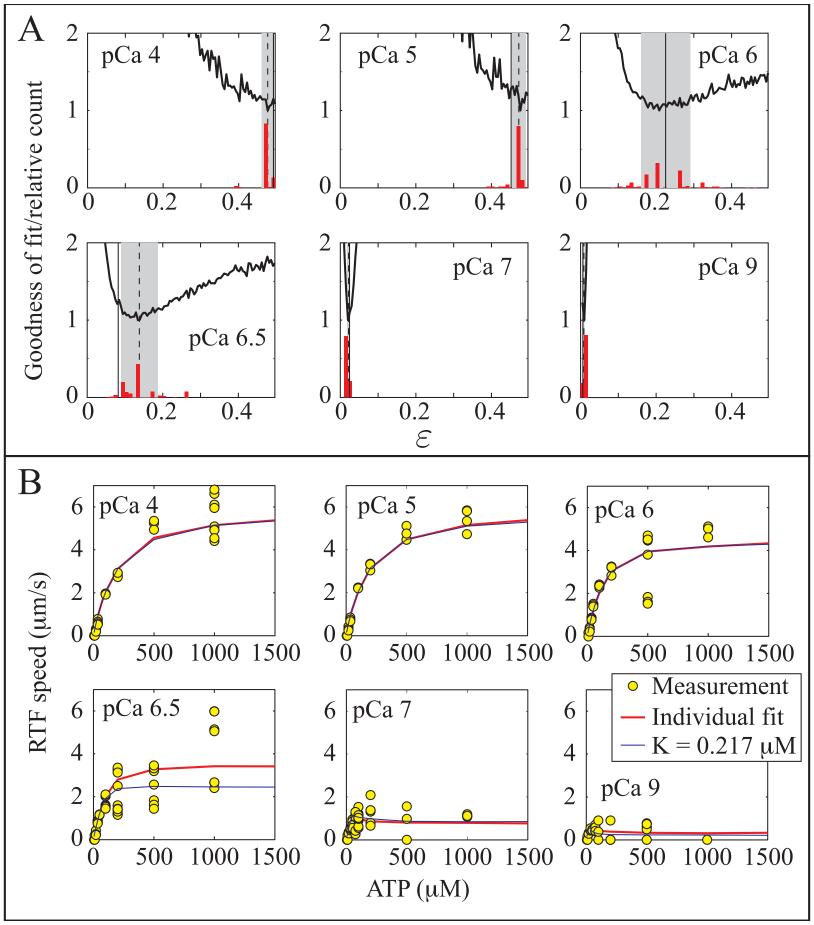

Figure S9: Estimating K from in vitro motility fits. A. Estimating $\varepsilon(\mathrm{Ca}^{++})$ from fitting in vitro motility data. Goodness-of-fit (solid curve) is the sum of the squared error, normalized to be 1 at the minimum. Best-fit values of ε, calculated by bootstrapping, are shown in the red histograms. Best-fit values of ε (dashed line) plus/minus one standard deviation (shaded region) are shown along with the predictedε from the best-fit value of $K = 0.217\mu M$ (sold vertical line). B. The motility data agrees with both individual best fits and fits with $K = 0.217\mu M$.

(1000 μM).

2.3 Support for modeling assumptions

Analysis of the data depends on three assumptions of the model. First, we assume that the coupling distance $\ell = 400$nm is independent of calcium. Second, we assume a form for ε, given by Eq. 3. Third, we assume Michaelis-Menten saturation of Tn with Ca^{++}, given by Eq. 4. These assumptions can be justified by the fact that, with these assumptions, the model gives estimates of K that are consistent with our direct measurements. There is also additional evidence that supports these assumptions; here we discuss this additional evidence.

2.3.1 Coupling distance $\ell = 400$nm

Our estimate of $\ell = 400$ nm comes from previous work fitting motility data at pCa 8, and also estimating the myosin spacing at which a thin filament becomes activated in the laser trap ([8] fitting data from [6]). With this value, the model describes motility in the presence of myosin binding protein-C at high and low calcium [10] and, assuming that it does not depend on Ca^{++}, the model describes observations of myosin

molecules binding to RTF in solution [11]. Besides this previous work, our current data also provides evidence of a coupling distance $\ell = 400$ nm.

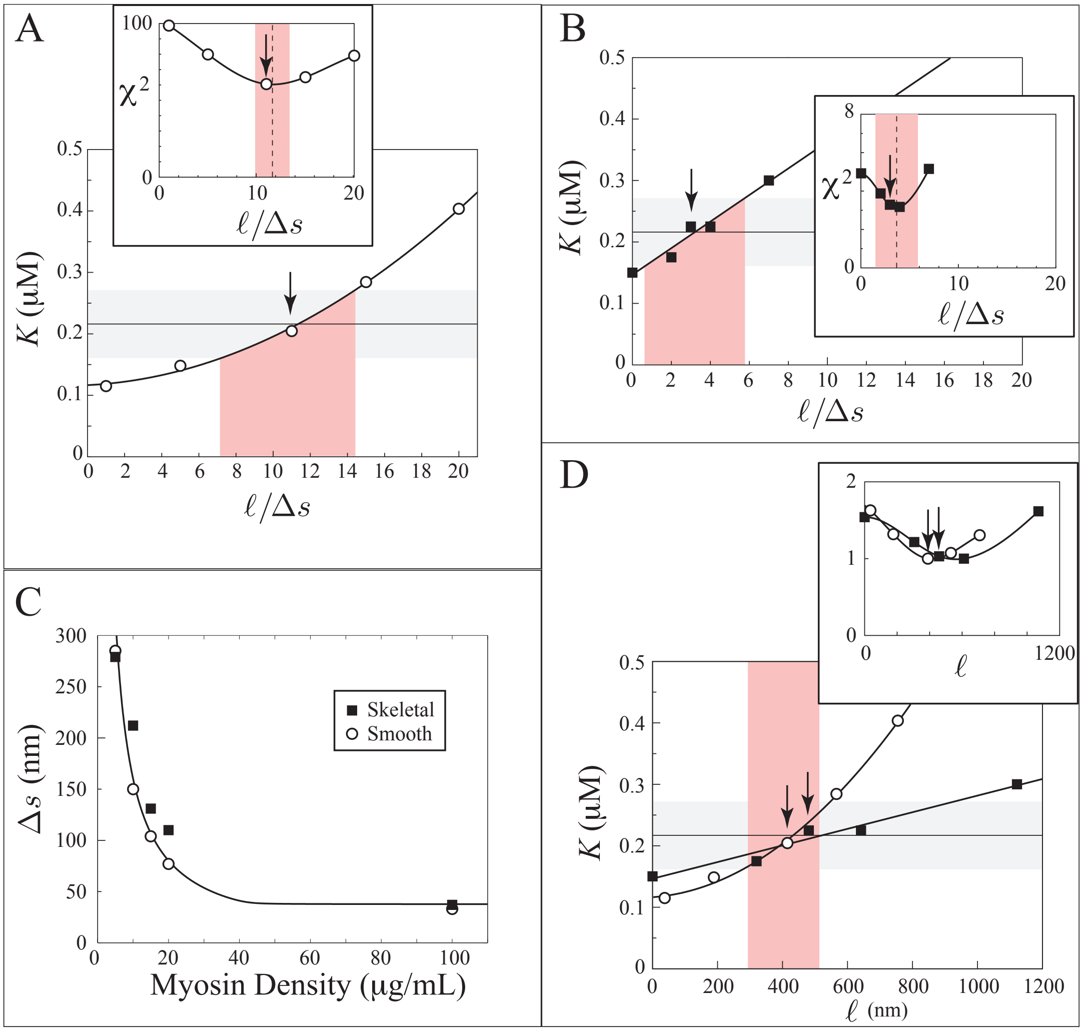

Figure S10: Our mini-ensemble and in vitro motility measurements independently support a coupling distance of $\ell = 400$ nm. In all plots, Δs is the spacing between myosin molecules along the RTF. The values of $\ell/\Delta s$ used in our simulations (11 for motility, 3 for mini-ensemble) are indicated with an arrow. A. A plot of the best-fit value of K as a function of molecules coupled ($\ell/\Delta s$) is only consistent with the best-fit value of K from our single molecule measurements (solid line, best fit, shaded area, plus/minus one quartile) for $\ell/\Delta s = 11 \pm 3$ (pink region). Fixing $K = 0.216\mu$M, the best-fit value from our single molecule measurements, identifies the same best-fit, but a tighter range ($\ell/\Delta s = 11 \pm 1$, pink region, inset). B. A plot of the best-fit value of K as a function of molecules coupled ($\ell/\Delta s$) is only consistent with the best-fit value of K from our single molecule measurements (solid line, best fit, shaded area, plus/minus one quartile) for $\ell/\Delta s = 3 \pm 2$ (pink region). Fixing $K = 0.216\mu$M, the best-fit value from our single molecule measurements, identifies a similar best-fit and range ($\ell/\Delta s = 4 \pm 2$, pink region, inset). C. An estimate of spacing between myosin molecules along a RTF as a function of myosin density (data from Harris and Warshaw 1993), with a fit from Eq. 5. From this curve, we get an estimate of $\Delta s = 35$ nm in motility and $\Delta s = 153$ nm in the mini ensemble experiments. D. Using the different values of Δs to re-plot the curves from A and B as a function of ℓ, shows a consistent best-fit value of $\ell = 400 \pm 100$ nm.

Varying ℓ changes the importance of myosin strong binding activation; a smaller ℓ means less myosin activation, a larger ℓ means more. Thus, if we underestimate ℓ, the model should predict too much Ca^{++} activation (i.e. underestimate K); if we overestimate ℓ, the model should predict too little Ca^{++} activation (i.e. overestimate K). We can therefore determine the optimum value of ℓ by performing a series of fits,

with variable ℓ, and seeing which value gives best agreement with our direct single molecule measurement of K.

The model does not directly depend on ℓ, but rather depends on $\ell/\Delta s$, where Δs is the spacing between myosin molecules (Eq. 2). This non-dimensional parameter gives the number of coupled myosin molecules. For the simulations of motility, we re-did our estimate of the best fit K (as described in section 2.2.3 In vitro motility) for $\ell/\Delta s = 1, 5, 15$, and 20 (we used $\ell/\Delta s = 11$ in that section). For the small ensemble trap simulations, we re-did our estimate of the best fit K (as described in section 2.2.2 Mini-ensemble laser trap) for $\ell/\Delta s = 0, 2, 4$, and 7 (we used $\ell/\Delta s = 3$ in that section).

In both simulations, as expected, the best-fit value of K increased with increasing ℓ. Using our single molecule estimate of $K = 0.216 \pm 0.055\mu$M identifies a range of possible values of $\ell/\Delta s$, giving $\ell/\Delta s = 11 \pm 3$ for motility and $\ell/\Delta s = 3 \pm 2$ (Fig. S10A,B). As an alternate means to estimate ℓ, we fixed $K = 0.216\mu$M and compared the model to the data for each value of $\ell/\Delta s$. This procedure identified $\ell/\Delta s = 11 \pm 1$ and $\ell/\Delta s = 4 \pm 2$ for motility and small ensemble measurements, respectively (Fig. S10A,B, insets) consistent with our previous estimates.

To estimate ℓ, we need an estimate of Δs for the two experiments. Harris and Warshaw (1993) [26] provide an estimate for this value as a function of myosin density in solution, M (in μg/mL). To obtain the best estimate for Δs, we fit these measurements with the following equation

$$\Delta s = \frac{2 M_{sat} \Delta s_{min}}{\left((M + M_{sat} + K_{eq}) - \sqrt{(M + M_{sat} + K_{eq})^2 - 4 * M_{sat} M}\right)} \tag{5}$$

where the parameters M_{sat} is the myosin concentration (in μg/mL) at which the surface becomes saturated, $K_{eq} = k^-/k^+$ is the equilibrium constant for unbinding from the surface (i.e. k^- is the rate myosin unbinds from the surface in s^{-1} and k^+ is the rate myosin binds to the surface in $s^{-1}(\mu g/mL)^{-1}$) and Δs_{min} is the spacing between myosin molecules when the surface is saturated. This equation comes from the steady-state of the following reaction mechanism, which assumes that there are a discrete and finite number of binding sites on the flow cell surface to which myosin can attach [27]

$$M + B \underset{k^-}{\overset{k^+}{\rightleftharpoons}} MB$$

where B is a binding site, M is a myosin molecule in solution and MB is a myosin molecule bound to the surface. Assuming relatively strong affinity of myosin for the surface, $K_{eq} = 0.1\mu$g/mL gives a reasonable fit to the data, with $M_{sat} = 43\mu$g/mL and $\Delta s_{min} = 35$ nm (Fig. S10C).

Given this estimate of Δs, we can transform our estimates of $\ell/\Delta s$ for the motility and mini ensemble laser trap measurements to a common scale of ℓ. The data are all remarkably consistent, with the estimate of K from our single molecule, mini-ensemble and motility measurements all intersecting near $K = 0.2\mu$M and $\ell = 400$ nm (Fig. S10D). More quantitatively, in order to explain each of our three measurements, the model requires a coupling distance of $\ell = 400 \pm 100$ nm. If the coupling is weaker than that, then the motility data and the small ensemble laser trap data require more Ca^{++} activation than we observed in our direct measurements; if the coupling is stronger than that, then the motility data and small ensemble laser trap data require less Ca^{++} activation than we observed. With $K = 0.216\mu$M, the best-fit to both data sets was also near $\ell = 400$nm (Fig. S10D, inset). Thus, myosin strong-binding activation, with $\ell = 400$ nm, is both necessary and sufficient to explain both the mini ensemble and motility measurements.

2.4 Cellular-scale simulations

We simulated isometric force as a function of pCa for a skinned muscle fiber. To do so, we ran simulations of 300 myosin molecules, with $\ell/\Delta s = 11$, the same coupling as in the motility assay. In each simulation,

at each pCa value, we used a modified Gillespie algorithm to perform 15,000 chemical reactions (myosin binding, ADP hydrolysis, unbinding, ATP hydrolysis) for the entire ensemble. We assumed an ATP concentration of 4mM. Since we are interested in isometric force, we kept the RTF stationary throughout the simulation, and kept track of the aggregate force produced by all attached motors ($F(t)$). If the simulation started at time $t = 0$ and ended at time $t = T$, we determined average force by interpolating $F(t)$ with 1001 points equally spaced between time $t = 0.25T$ and T. In this way, we get a time averaged force (i.e. approximate $\frac{1}{0.75T}\int_{0.25T}^{T} F(t)dt$), and neglect non steady-state effects.

2.4.1 Force-pCa parameters

We estimated fitting parameters for isometric force as a function of pCa, in order to compare our model to the measurements of Millar and Homsher 1990 [28]. We ran cellular-scale simulations at each pCa value used by Millar and Homsher 1990 [28], pCa 8, 7.5, 7, 6.8, 6.7, 6.5, 6.3, 6, 5.5, and 4.5. We ran 10 simulations at each pCa.

We calculated the best fit by minimizing the mean squared difference between our simulations and the following Hill model

$$F([\mathrm{Ca}^{++}]) = F_0 + \Delta F \frac{[\mathrm{Ca}^{++}]^{\alpha}}{[\mathrm{Ca}^{++}]^{\alpha} + K_I^{\alpha}}$$

where $[\mathrm{Ca}^{++}] = 10^{-pCa}\mathrm{M}$, F_0 is the force generated in the absence of Ca^{++}, ΔF is the difference between force at maximal activation and F_0, α is the Hill coefficient, and $K_I = 10^{-pCa_{50}}$. We assumed that F_0 is the average of the 10 isometric force simulations at pCa 8, the remaining parameters (ΔF, α and pCa_{50}) were determined from the best fit. To estimate the error associated with these best fit parameters, we used a bootstrapping approach. At each pCa concentration, we randomly selected from our 10 simulations, with replacement, until we had a full complement of 10 values. We then fit the resulting data, using Matlab's fminsearch function (a Nelder-Mead simplex algorithm) with an initial random seed to find the values of α, pCa_{50} and ΔF that minimize mean squared error. We repeated this process 10,000 times, giving a distribution of best-fit values. In this way, we estimate $\alpha = 1.71 \pm 0.19$ and $\mathrm{pCa}_{50} = 6.81 \pm 0.03$ (means plus/minus standard deviation). Note that these values are insensitive to our assumed number of myosin molecules in a half sarcomere, provided that there are a sufficiently large number, since with an ensemble of 150 molecules we found $\alpha = 1.65 \pm 0.20$ and $\mathrm{pCa}_{50} = 6.81 \pm 0.03$, and with an ensemble of 75 molecules we found $\alpha = 1.58 \pm 0.15$ and $\mathrm{pCa}_{50} = 6.79 \pm 0.03$.

2.4.2 Binding of Ca^{++} to Tn

To further explore if and how strong binding activates the RTF, we used the model to predict how Tn saturates with Ca^{++}, both in the presence and absence of myosin strong binding. If strong binding leads to RTF activation, and if Ca^{++} binding to Tn is proportional to RTF activation, then measurements of Tn saturation as a function of Ca^{++} in the presence of myosin strong binding should show evidence of increased Ca^{++} sensitivity (i.e. a leftward shift). Indeed this effect has been observed in muscle fibers [29, 30], but it remains controversial because others have not seen this shift [31, 32]. By predicting these measurements, the model can provide independent support for one of these two contradictory observations.

In the model, without myosin strong binding, the Tn saturation curve is given by equation (4), a Michaelis-Menten curve with a Michaelis-Menten constant of $K = 0.2\mu\mathrm{M}$. This gives half Tn saturation at pCa 6.7, nearly identical to the isometric force $\mathrm{pCa}_{50} = 6.8$. This prediction can be tested by a previously published measurement of Ca^{++} binding to Tn in muscle fibers stretched to where the thick and thin filaments barely overlap (an average sarcomere length of $3.8\mu\mathrm{m}$) [29]. When plotted relative to the pCa_{50} of the isometric force-Ca^{++} curve, the measured Tn saturation curve agrees well with the prediction of our model. Indeed, the two are not significantly different (Fig. S11A, $p > 0.05$, χ^2 test).

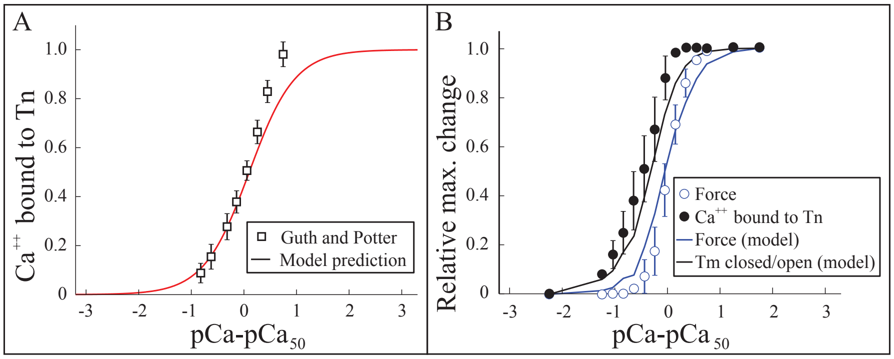

Figure S11: Model predicts a myosin-induced increase in the binding of Ca^{++} to Tn. A. Model predictions of Ca^{++} binding to troponin (Tn) in the absence of myosin strong binding (solid line), given by equation (4), agree with cellular measurements [29] (squares, error bars show SEM.). B. Model predictions of a leftward shift in Ca^{++} binding to Tn in the presence of myosin strong binding (black line), which indicates strong-binding induced activation, are consistent with some experimental measurements [30] (black symbols, error bars show SEM.). The horizontal axis shows pCa relative to the pCa_{50} of isometric force

To predict Tn saturation in the presence of strong binding, we assume that myosin strong binding transitions Tn into a state with high Ca^{++} affinity. Then, the model predicts that myosin strong binding induces a leftward shift of 0.27 ± 0.07 pCa (mean plus/minus SD, Fig. S11B). Measurements of this value vary, depending on ionic strength and Mg^{++} concentration used in the experimental solutions, but previously observed values are 0.28 pCa [29] (200mM ionic strength), 0.48 ± 0.11 [30] (1mM Mg^{++}) and 0.21 ± 0.08 [30] (5mM Mg^{++}). The model successfully predicts the existence and the magnitude of this shift. Therefore, by incorporating our measurements into a model, we add independent support to the hypothesis that myosin strong binding to a RTF activates it and leads to Ca^{++} binding to Tn [29, 30], even at physiological ATP concentrations.

2.4.3 Functional form of $\varepsilon(Ca^{++})$

We have assumed a particular form of $\varepsilon(Ca^{++})$, given by Eq. 3. This form is based on the assumption that myosin can always bind to a RTF, regardless of the state of Tn or Tm, but the energy required to do so varies depending on Tn's saturation with Ca^{++}. This assumption leads to the prediction that attachment rate varies exponentially with Tn's saturation with Ca^{++}, as opposed to assuming that attachment rate varies in proportion to Tn's saturation with Ca^{++}.

If attachment rate varies in proportion to Tn's saturation with Ca^{++} then, in the absence of myosin's interaction with actin, Tn should be half saturated around pCa 6 (based on our measurements of $\varepsilon(Ca^{++})$). Alternatively, if attachment rate varies exponentially with Tn's saturation with Ca^{++} then, in the absence of myosin's interaction with actin, Tn should be half saturated around pCa 6.7 (the negative log base 10 of K). Under isometric conditions, half maximal force is produced around pCa 6.8. Thus, the proportional model predicts that Tn reaches half saturation *at a much lower pCa value* as half maximal force, while the exponential model predicts that Tn reaches half saturation *approximately at the same pCa value* as half maximal force. Fig. S11A shows that, in the absence of myosin interacting with actin, Tn half saturation and half maximal force occur at the same pCa value [29]. Thus, these data support our assumed form of Eq. 3.

2.4.4 Michaelis-Menten binding of Ca^{++} to Tn

We have assumed Michaelis-Menten binding of Ca^{++} to Tn; however, since there are two primary Ca^{++}-binding sites on skeletal Tn, one might anticipate cooperative binding. We investigated whether we can

see evidence of cooperative binding in two ways.

First, since our estimate of $\varepsilon(\text{Ca}^{++})$ from our in vitro motility measurements was independent of Eqs. 3 and 4, we fit this estimate with a Hill model

$$\theta = \frac{[\text{Ca}^{++}]^{\alpha}}{K_H^{\alpha} + [\text{Ca}^{++}]^{\alpha}} \tag{6}$$

where α is a cooperativity parameter (of course, Eq. 4 is a special case of this equation with $\alpha = 1$). If a Hill model fits these data significantly better than Eq. 4, then we have evidence of cooperative Ca^{++} binding to Tn. However, the best fit of Eq. 6 (with $\alpha = 1.171$ and $K_H = 0.190\mu$M) was not significantly different from the Michaelis-Menten fit ($p > 0.05$, F-test).

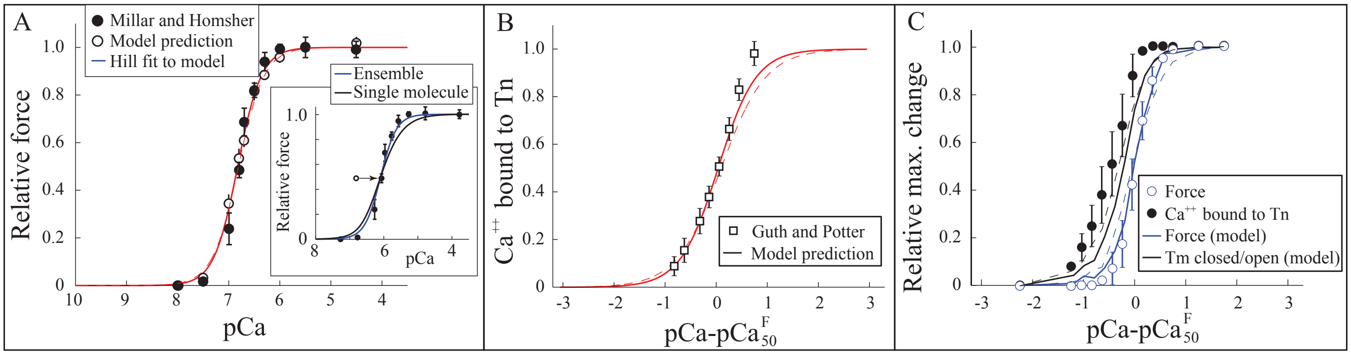

Figure S12: Adding cooperativity to our model of Ca^{++} binding to Tn has only a small effect on predictions at the fiber scale. In all plots, the best Michaelis-Menten fit (Eq. 4) is shown as a dashed line; the best Hill fit (Eq. 6, with $\alpha = 1.171$) is shown as a solid line. Data (symbols, error bars SEM) are the same as in Fig. 5 of the main text. A. Isometric force as a function of pCa (inset shows the increase in slope due to myosin binding activation). B. Ca^{++} binding to Tn in the absence of myosin binding. The model prediction from the Hill fit is significantly better than the model prediction from the Michaelis-Menten fit (F-test, $p < 0.05$). C. A leftward shift in Ca^{++} binding to Tn in the presence of myosin strong binding. In B and C, the horizontal axis shows pCa relative to the pCa_{50} of isometric force.

Second, the measurements of Güth an Potter (1987) [29] of Ca^{++} binding to Tn in the absence of myosin's interaction with actin are well-predicted by the model (prediction and data not significantly different, $p > 0.05$, χ^2 test). Interestingly, however, the best Hill fit to the motility data was a significantly better fit to these data than the best Michaelis-Menten fit (Fig. S12B, $p < 0.05$ F-test). Thus, these data are consistent with weak cooperativity ($\alpha = 1.171$). Predictions of isometric force (Fig. S12A) and Ca^{++} binding to Tn in the presence of myosin strong binding (Fig. S12C) are qualitatively the same as the best Michaelis-Menten fit (i.e. we see evidence of strong binding activation, including an increase in slope of the isometric force-pCa curve (Fig. S12A), and a left-ward shift of Tn's binding of Ca^{++} (Fig. S12C)), but show slightly better agreement with the data. Thus, the fiber data suggest that weak cooperativity exists in Tn's binding of Ca^{++}; however, it is worth noting that fiber force data includes contributions from non-myosin, Ca^{++}-sensitive proteins, like titin [33] and myosin binding protein C [34, 35]. Thus, it is hard to know whether differences between these measurements and the model's predictions arise because of weak Tn cooperativity or from the force generation of these accessory proteins.

Thus, our own data do not allow us to conclude that cooperativity occurs. Fiber data, which might include non-myosin force generation, are consistent with weak cooperativity. Therefore, in this study, we assume no cooperativity, with the additional justification that it allows us to get rid of one parameter (i.e. we don't need to specify α).

References

[1] S. C. Pastra-Landis, T. Huiatt, and S. Lowey. Assembly and kinetic properties of myosin light chain isozymes from fast skeletal muscle. *J Mol Biol*, 170:403–422, 1983.

[2] M. Kovács, J. Tóth, C. Hetényi, A. Málnási-Csizmadia, and J. R. Sellers. Mechanism of blebbistatin inhibition of myosin II. *J Biol Chem*, 279:35557–35563, 2004.

[3] M. Wu, J. W. Roberts, and M. Buckley. Three dimensional fluorescent particle tracking at micron-scale using a single camera. *Experiments in Fluids*, 38:461–465, 2005.

[4] A. E. Knight, C. Veigel, C. Chambers, and J. E. Molloy. Analysis of single-molecule recordings: applications to acto-myosin recordings. *Prog Biophys Mol Biol*, 177:45–72, 2001.

[5] M. J. Tyska, D. E. Dupuis, W. H. Guilford, J. B. Patlak, G. S. Waller, K. M. Trybus, D. M. Warshaw, and S. Lowey. Two heads of myosin are better than one for generating force and motion. *Proc Natl Acad Sci*, 96:4402–4407, 1999.

[6] N. M. Kad, S. Kim, D. M. Warshaw, P. VanBuren, and J. E. Baker. Single-myosin crossbridge interactions with actin filaments regulated by troponin-tropomyosin. *Proc Natl Acad Sci*, 102:16990–16995, 2005.

[7] S. Walcott, D. M. Warshaw, and E. P. Debold. Mechanical coupling between myosin molecules causes differences between ensemble and single-molecule measurements. *Biophys J*, 103:501–510, 2012.

[8] S. Walcott. A differential equation model for tropomyosin-induced myosin cooperativity describes myosin-myosin interactions at low calcium. *Cell Mol Bioeng*, 6:13–25, 2013.

[9] S. Walcott. Muscle activation described with a differential equation model for large ensembles of locally coupled molecular motors. *Phys Rev E*, 90:042717, 2014.

[10] S. Walcott, S. Docken, and S. P. Harris. Effects of cardiac myosin binding protein-c on actin motility are explained with a drag-activation-competition model. *Biophys J*, 108:10–13, 2015.

[11] S. Walcott and N. M. Kad. Direct measurements of local coupling between myosin molecules are consistent with a model of muscle activation. *PLoS Comput Biol*, 11:e1004599, 2015.

[12] C. Veigel, J. E. Molloy, S. Schmitz, and J. Kendrick-Jones. Load-dependent kinetics of force production by smooth muscle myosin measured with optical tweezers. *Nat. Cell Bio.*, 5:980–986, 2003.

[13] N. M. Kad, J. B. Patlak, P. M. Fagnant, K. M. Trybus, and D. M. Warshaw. Mutation of a conserved glycine in the sh1-sh2 helix affects the load-dependent kinetics of myosin. *Biophys. J.*, 92:1623–1631, 2007.

[14] C. Veigel, M. L. Bartoo, D. C. White, J. C. Sparrow, and J. E. Molloy. The stiffness of rabbit skeletal actomyosin cross-bridges determined with an optical tweezers transducer. *Biophys J*, 75:1424–1438, 1998.

[15] E. P. Debold, S. Walcott, M. Woodward, and M. A. Turner. Direct observation of phosphate inhibiting the force-generating capacity of a mini ensemble of myosin molecules. *Biophys J*, 105:2374–2384, 2013.

[16] M. Capitanio, M. Canepari, P. Cacciafesta, V. Lombardi, R. Cicchi, M. Maffei, F. S. Pavone, and R. Bottinelli. Two independent mechanical events in the interaction cycle of skeletal muscle myosin with actin. *Proc. Natl. Acad. Sci.*, 103:87–92, 2006.

[17] M. J. Greenberg, H. Shuman, and E. M. Ostap. Inherent force-dependent properties of ?-cardiac myosin contribute to the force-velocity relationship of cardiac muscle. *Biophys J*, 107:L41–L44, 2014.

[18] J. Sung, S. Nag, K. I. Mortensen, C. L. Vestergaard, S. Sutton, K. Ruppel, H. Flyvbjerg, and J. A. Spudich. Harmonic force spectroscopy measures load-dependent kinetics of individual human β-cardiac myosin molecules. *Nat commun*, 6:7931, 2015.

[19] D. A. Smith. Path-integral theory of an axially confined worm-like chain. *J Phys A: Math Gen*, 34:4507, 2001.

[20] D. A. Smith, R. Maytum, and M. A. Geeves. Cooperative regulation of myosin-actin interactions by a continuous flexible chain I: actin-tropomyosin systems. *Biophys J*, 84:3155–3167, 2003.

[21] D. A. Smith and M. A. Geeves. Cooperative regulation of myosin-actin interactions by a continuous flexible chain II: actin-tropomyosin-troponin and regulation by calcium. *Biophys J*, 84:3168–3180, 2003.

[22] J. Howard. *Mechanics of motor proteins and the cytoskeleton*. Sinauer, 2001.

[23] D. F. McKillop and M. A. Geeves. Regulation of the interaction between actin and myosin subfragment 1: evidence for three states of the thin filament. *Biophys J*, 65:693–701, 1993.

[24] D. T. Gillespie. Exact stochastic simulation of coupled chemical reactions. *J Phys Chem*, 81:2340–2361, 1977.

[25] M. J. Greenberg, C. L. Wang, W. Lehman, and J. R. Moore. Modulation of actin mechanics by caldesmon and tropomyosin. *Cell Motil Cytoskeleton*, 65:156–164, 2008.

[26] D. E. Harris and D. M. Warshaw. Smooth and skeletal muscle myosin both exhibit low duty cycles at zero load in vitro. *J Biol Chem*, 268:14764–14768, 1993.

[27] S. Walcott, P. M. Fagnant, K. M. Trybus, and D. M. Warshaw. Smooth muscle myosin phosphorylated on one of its two heads supports force and motion. *J Biol Chem*, 284:18244–18251, 2009.

[28] N. C. Millar and E. Homsher. The effect of phosphate and calcium on force generation in glycerinated rabbit skeletal muscle fibers. a steady-state and transient kinetic study. *J Biol Chem*, 265:20234–20240, 1990.

[29] K. Güth and J. D. Potter. Effect of rigor and cycling cross-bridges on the structure of troponin C and the Ca^{2+} affinity of the Ca^{2+}-specific regulatory sites in skinned rabbit psoas fibers. *J Biol Chem*, 262:13627–13635, 1987.

[30] B. Parsons, D. Szczesna, J. Zhao, G. van Slooten, W. G. L. Kerrick, J. A. Putkey, and J. D. Potter. The effect of ph on the Ca^{2+} affinity of the Ca^{2+} regulatory sites of skeletal and cardiac troponin C in skinned muscle fibres. *J Musc Res Cell Motil*, 18:599–609, 1997.

[31] D. A. Martyn, C. J. Freitag, P. B. Chase, and A. M. Gordon. Ca^{2+} and cross-bridge-induced changes in troponin C in skinned skeletal muscle fibers: effects of force inhibition. *Biophys J*, 76:1480–1493, 1999.

[32] D. A. Martyn and A. M. Gordon. Influence of length on force and activation-dependent changes in troponin C structure in skinned cardiac and fast skeletal muscle. *Biophys J*, 80:2798–2808, 2001.

[33] R. Tatsumi, K. Maeda, A. Hattori, and K. Takahashi. Calcium binding to an elastic portion of connectin/titin filaments. *J Muscle Res Cell Motil*, 22:149–162, 2001.

[34] M. V. Razumova, J. F. Shaffer, A.-Y. Tu, G. V. Flint, M. Regnier, and S. P. Harris. Effects of the n-terminal domains of myosin binding protein-c in an in vitro motility assay: evidence for long-lived cross-bridges. *J. Biol. Chem.*, 281:35846–35854, 2006.

[35] M. J. Previs, B. L. Prosser, J. Y. Mun, S. B. Previs, J. Gulick, K. Lee, J. Robbins, R. Craig, W. J. Lederer, and D. M. Warshaw. Myosin-binding protein c corrects an intrinsic inhomogeneity in cardiac excitation-contraction coupling. *Sci. Adv.*, 1:e1400205, 2015.
